# Structural biology and functional features of phage-derived depolymerase Depo32 on *Klebsiella pneumoniae* with K2 serotype capsular polysaccharides

**DOI:** 10.1128/spectrum.05304-22

**Published:** 2023-09-26

**Authors:** Ruopeng Cai, Zhuolu Ren, Rihong Zhao, Yan Lu, Xinwu Wang, Zhimin Guo, Jinming Song, Wentao Xiang, Rui Du, Xiaokang Zhang, Wenyu Han, Heng Ru, Jingmin Gu

**Affiliations:** 1 College of Animal Science and Technology, Jilin Agricultural University, Changchun, Jilin, China; 2 College of Veterinary Medicine, Jilin University, Changchun, Jilin, China; 3 Zhejiang Provincial Key Laboratory for Cancer Molecular Cell Biology, Life Sciences Institute, Zhejiang University, Hangzhou, Zhejiang, China; 4 College of Animal Science and Technology, Jilin Agricultural Science and Technology University, Changchun, Jilin, China; 5 Infectious Diseases and Pathogen Biology Center, Clinical Laboratory Department, First Hospital of Jilin University, Changchun, Jilin, China; 6 College of Chinese Medicine Materials, Jilin Agricultural University, Changchun, Jilin, China; 7 Faculty of Life and Health Sciences, Shenzhen Institute of Advanced Technology, Chinese Academy of Sciences, Shenzhen, Guangdong, China; 8 Inter disciplinary Center for Brain Information, The Brain Cognition and Brain Disease Institute, Shenzhen Institute of Advanced Technology, Chinese Academy of Sciences, Shenzhen, Guangdong, China; 9 Shenzhen-Hong Kong Institute of Brain Science-Shenzhen Fundamental Research Institutions, Shenzhen, Guangdong, China; 10 Jiangsu Co-Innovation Center for the Prevention and Control of Important Animal Infectious Diseases and Zoonoses, Yangzhou University, Yangzhou, Jiangsu, China; The University of Tennessee Knoxville, Knoxville, Tennessee, USA

**Keywords:** depolymerase, *Klebsiella pneumoniae*, K2 capsular type, structural biology, therapeutic effect

## Abstract

Hypervirulent *Klebsiella pneumoniae* with capsular polysaccharides (CPSs) causes severe nosocomial- and community-acquired infections. Phage-derived depolymerases can degrade CPSs from *K. pneumoniae* to attenuate bacterial virulence, but their antimicrobial mechanisms and clinical potential are not well understood. In the present study, *Klebsiella* phage GH-K3-derived depolymerase Depo32 (encoded by gene *gp32*) was identified to exhibit high efficiency in specifically degrading the CPSs of K2 serotype *K. pneumoniae*. The cryo-electron microscopy structure of trimeric Depo32 at a resolution up to 2.32 Å revealed potential catalytic centers in the cleft of each of the two adjacent subunits. *K. pneumoniae* subjected to Depo32 became more sensitive to phagocytosis by RAW264.7 cells and activated the cells by the mitogen-activated protein kinase signaling pathway. In addition, intranasal inoculation with Depo32 (a single dose of 200 µg, 20 µg daily for 3 days, or in combination with gentamicin) rescued all C57BL/6J mice infected with a lethal dose of *K. pneumoniae* K7 without interference from its neutralizing antibody. In summary, this work elaborates on the mechanism by which Depo32 targets the degradation of K2 serotype CPSs and its potential as an antivirulence agent.

**IMPORTANCE:**

Depolymerases specific to more than 20 serotypes of *Klebsiella* spp. have been identified, but most studies only evaluated the single-dose treatment of depolymerases with relatively simple clinical evaluation indices and did not reveal the anti-infection mechanism of these depolymerases in depth. On the basis of determining the biological characteristics, the structure of Depo32 was analyzed by cryo-electron microscopy, and the potential active center was further identified. In addition, the effects of Depo32 on macrophage phagocytosis, signaling pathway activation, and serum killing were revealed, and the efficacy of the depolymerase (single treatment, multiple treatments, or in combination with gentamicin) against acute pneumonia caused by *Klebsiella pneumoniae* was evaluated. Moreover, the roles of the active sites of Depo32 were also elucidated in the *in vitro* and *in vivo* studies. Therefore, through structural biology, cell biology, and *in vivo* experiments, this study demonstrated the mechanism by which Depo32 targets K2 serotype *K*. *pneumoniae* infection.

## INTRODUCTION

As common zoonotic pathogens, most *Klebsiella* spp. have the ability to synthesize and secrete capsular polysaccharides (CPSs). As the major virulence factors of pathogenic microorganisms, CPSs are associated with the maintenance of bacterial virulence, adherence, biofilm formation, and evasion from complement-mediated opsonophagocytosis by the host’s immune system, and they prevent the penetration of some antibiotics into strains (1). Thus far, at least 80 serotypes (K-antigens) and 163 capsular locus types have been recognized in *Klebsiella* spp. (2 – 4). K1, K2, K5, K54, and K57 are most frequently associated with invasive disease or the pathogenicity of *Klebsiella pneumoniae* (5). Compared with other capsular types, the K2 capsule structure lacks repeating D-mannose-a-2,3-D-mannose or L-rhamnose-a-2,3-L-rhamnose units, which can be recognized by the surface lectin of macrophages (6). Therefore, K2 serotype *K. pneumoniae* strains have been reported as hypervirulent strains due to evasion from host immunity (7).

Phage depolymerases specifically recognize the glycosidic bonds on the host surface and randomly degrade them to release the repeating units (oligosaccharides) of the glycopolymers; thus, the phage particles can move closer to the cell surface and bind to host receptors (8, 9). Depolymerases are divided into two major categories according to their catalytic mechanisms, namely, hydrolases and lyases. Hydrolases are enzymes that degrade glycosidic bonds by breaking the glycosyl-oxygen bond, and their subclasses include sialidases, levanases, xylosidases, dextranases, rhamnosidases, and peptidases. Lyases cleave the bonds between the monosaccharide and C4 uronic acid, and they introduce unsaturated bonds between C4 and C5 nonreducing uronic acid ends at the same time. Lyases include hyaluronidases, alginate lyases, and pectin/pectate lyases (10).

Some studies have indicated that depolymerases have the ability to remove bacterial biofilms and can be used to diagnose pathogens (11). In addition, depolymerases have shown potential in controlling fatal infections that are caused by some CPS-forming *K. pneumoniae* in both mouse models and *Galleria mellonella* models (12, 13). At present, depolymerases targeting CPSs of more than 20 serotypes of *K. pneumoniae* have been biochemically identified (14), but the antimicrobial mechanism of most of these depolymerases remains unclear.

**Fig 1:**
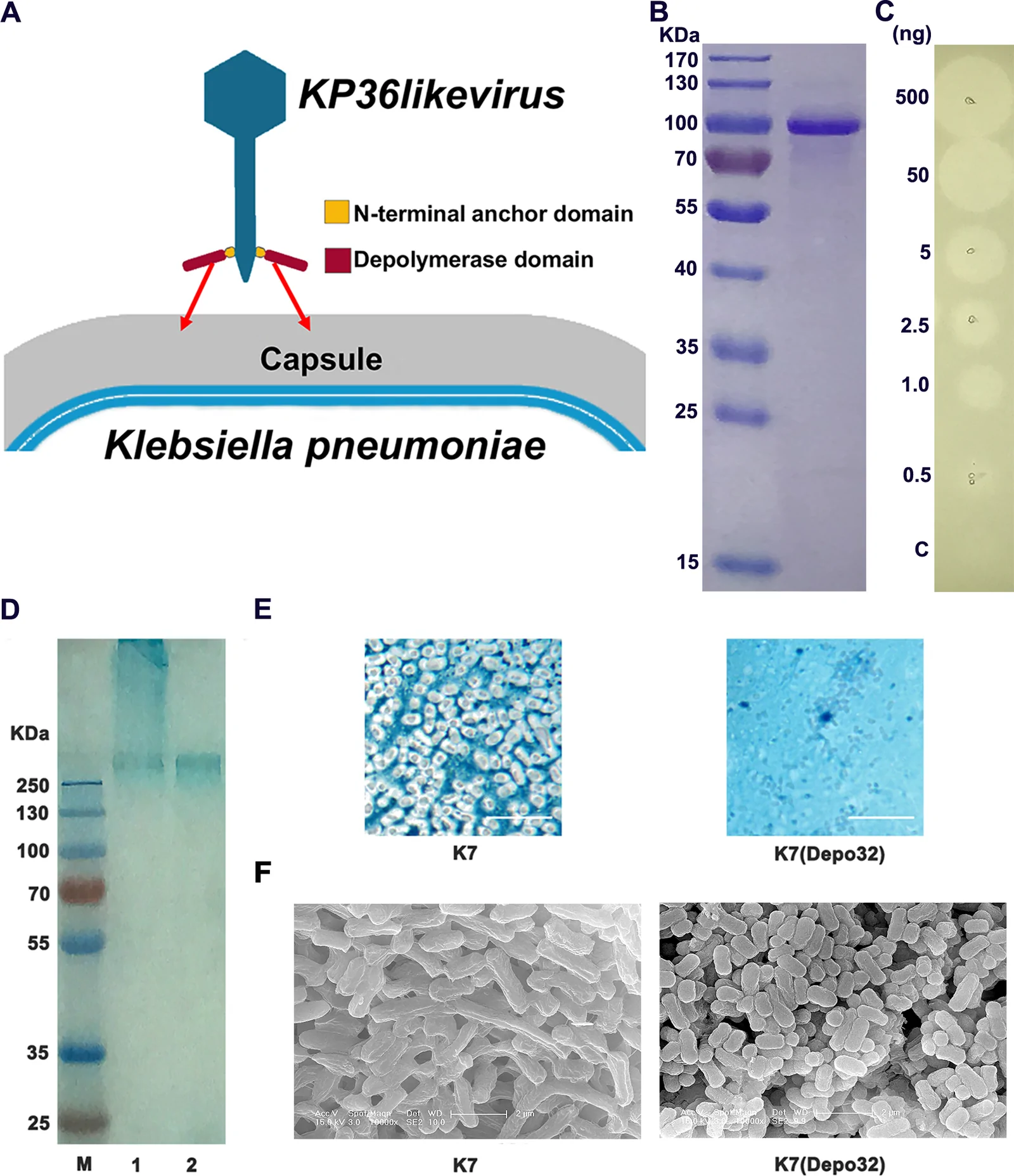
CPS degradation effect of Depo32 on *K. pneumoniae* K7. (**A**) Schematic model of the depolymerase with a conserved N-terminal anchor domain in phage particles of the “*KP36likevirus*” genus. (**B**) SDS-PAGE analysis. Purified Depo32 was separated by SDS-PAGE with Coomassie blue staining. The molecular weight (98 kDa) is indicated beside the protein markers. (**C**) Enzyme activity assay. Depo32 (100 µg/mL) solutions were serially diluted and spotted on lawns of *K. pneumoniae* K7 (0.5–500 ng). Tris buffer was used as a control. (**D**) CPS phenotypes. CPS samples were extracted from equal amounts of *K. pneumoniae* strains (1.0 × 10^9^ CFU). After separation by 12% SDS-PAGE, the CPS phenotypes of K7 (lane 1) and K7 (Depo32) (lane 2) were visualized by alcian blue staining. (**E**) Maneval’s staining. Maneval staining of the extracellular material of *K. pneumoniae* K7 (left) and K7(Depo32) (right) (magnification, ×1,000; scale bar, 10 µm). (**F**) Scanning electron microscopy. The surface morphology of *K. pneumoniae* K7 (left) and K7(Depo32) (right). Scale bars represent 2 µm.

Phages belonging to the “*KP36likevirus*” genus usually have a depolymerase-like protein with a similar N-terminal anchor domain (Fig. 1A) (8, 15). In our previous study, *Klebsiella* phage GH-K3 was classified as a member of the “*KP36likevirus*” genus, and gp32 of this phage was evaluated as a putative depolymerase-like protein (16). Here, GH-K3 *gp32* was identified to encode a depolymerase, Depo32, which efficiently degrades the CPSs of *K. pneumoniae* K7 (K2 capsular type). The cryo-EM structure, potential catalytic center, and active sites of Depo32 have been revealed. Furthermore, the promoting effect of Depo32 on macrophage endocytosis and serum killing, as well as the protective effect and the bacterial elimination ability of this depolymerase in mice infected with *K. pneumoniae*, has been clarified. Thus, the mechanism of Depo32 against K2 capsular type *K. pneumoniae* was elucidated, and the depolymerase exhibits great potential as an antivirulence and anti-infection agent.

## RESULTS

### Depo32 degrades the K2-type CPS of *K. pneumoniae*

In the SDS-PAGE analysis, a main band of approximately 98 kDa corresponding to the molecular weight of Depo32 was observed (Fig. 1B). Purified Depo32 was subsequently dropped onto a lawn of *K. pneumoniae* K7 after serial dilutions. The lower mass limit for Depo32 to form clear spots is 1.0 ng (Fig. 1C); thus, Depo32 was identified as a depolymerase with high activity. The pH tolerance range of Depo32 was 4–10 (Fig. S1A), and the temperature range of stable enzyme activity was 4–37 °C (Fig. S1B). High concentrations of EDTA (0.25 M) and NaCl (5 M) had a limited effect on Depo32 activity (Fig. S1C through D).

K7(Depo32) indicates that *K. pneumoniae* K7 was grown to exponential phase (OD_600_ ≈ 0.6–0.8) and then treated with Depo32 at 37°C for 3 h at a final concentration of 10 µg/mL. By alcian blue staining, the molecular weight of polysaccharides in K7(Depo32) was much lower than that of the CPS polymer of K7 (Fig. 1D). Maneval staining and microscopic analyses showed that the wild-type K7 strains were surrounded by thick capsules. After treatment with Depo32, the capsules were faint (Fig. 1E). In addition, SEM analyses confirmed that a drastic reduction in CPS occurred on the surface of K7(Depo32). The boundaries of these bacteria were clearer than those of nontreated cells (Fig. 1F). However, Depo32 only digested capsules of the K2 capsular type *K. pneumoniae* strains (KP1, KPP6, KPP7, KPP14, KPP27, KPP36, KPP41, and KPP51) (Fig. S2).

### Cryo-EM structure determination of Depo32 at atomic resolution

**Fig 2:**
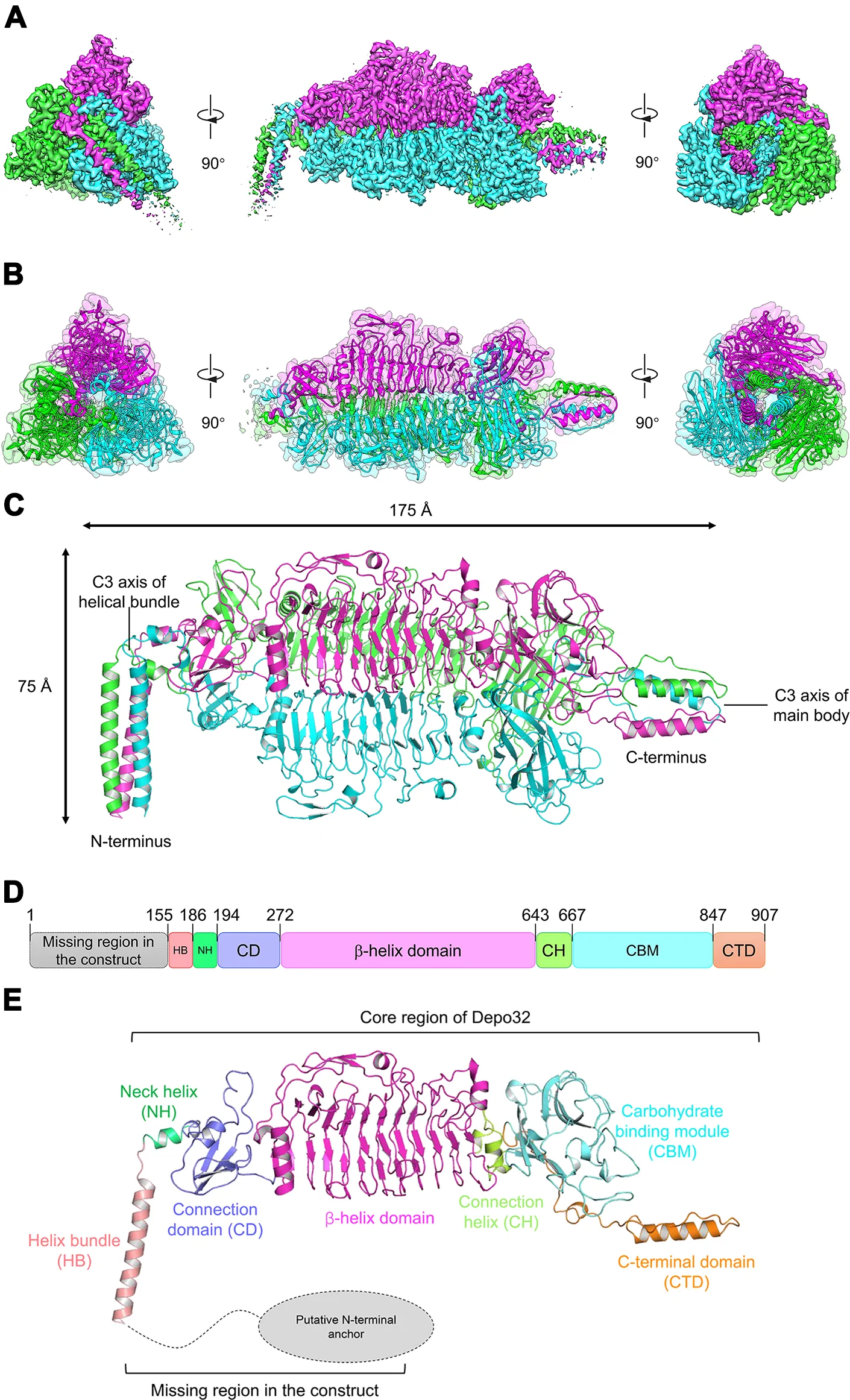
Overall structure and domain organization of Depo32. (**A**) Orthogonal view of the nonsymmetrized cryo-EM map of Depo32 at 2.46 Å resolution. From left to right, top, side, and bottom views. (**B**) Superposition of the C3-symmetrized cryo-EM map and the atomic model of Depo32 built into the 2.32 Å-resolution map. From left to right, top, side, and bottom views. (**C**) Atomic model of the Depo32 trimer built based on the 2.46 Å nonsymmetrized map. The trimer assumes an elongated spindle-like shape that is approximately 175 Å in length and 75 Å in diameter at the widest region. (**D**) Domain organization of Depo32. HB, helix bundle; NH, neck helix; CD, connection domain; CH, connection helix; CBM, carbohydrate-binding module; and CTD, C-terminal domain. (**E**) Cartoon representation of the Depo32 monomer structure colored based on panel (**D**), each domain is represented.

To understand the whole architecture as well as the potential modes of action of Depo32 on the K2-type CPS of *K. pneumoniae*, the structure of Depo32 was solved by cryo-electron microscopy (cryo-EM) (Fig. 2A; Fig. S3). Atomic models were built into the 2.46 Å nonsymmetrized and 2.32 Å C3-symmetrized maps and refined iteratively in real and reciprocal spaces (Fig. 2B; Fig. S4D and E; Table S1). Both the nonsymmetrized and C3-symmetrized maps presented well-defined electron densities in the core region, and they all revealed trimeric architectures, similar to the overall characteristics of the previously identified tailspike proteins with known structures (17, 18) (Fig. 2A through C; Fig. S5A). In addition to the invisible N-terminal anchor domain and the helical-bundle domain (to residue 185) that may form the head of the spike protein, the remaining core region of the Depo32 monomer can be further split into the following domains: (i) a very short neck helix and the connection domain (residues 186–271), (ii) the β-helix domain (residues 272–642), (iii) the connection helix domain (residues 643–666), (iv) the carbohydrate-binding module (CBM) (residues 667–846), and (v) a C-terminal domain (residues 847–907) (Fig. 2D and E; Fig. S5B).

In the core region of Depo32, since the resolution of the C3-symmetrized map is slightly higher than that of the nonsymmetrized map, and because of the inclusion of the potential CPS binding, catalytic and/or regulatory domains in this region, we then focused on the C3-symmetrized map and model to perform the subsequent analyses.

**Fig 3:**
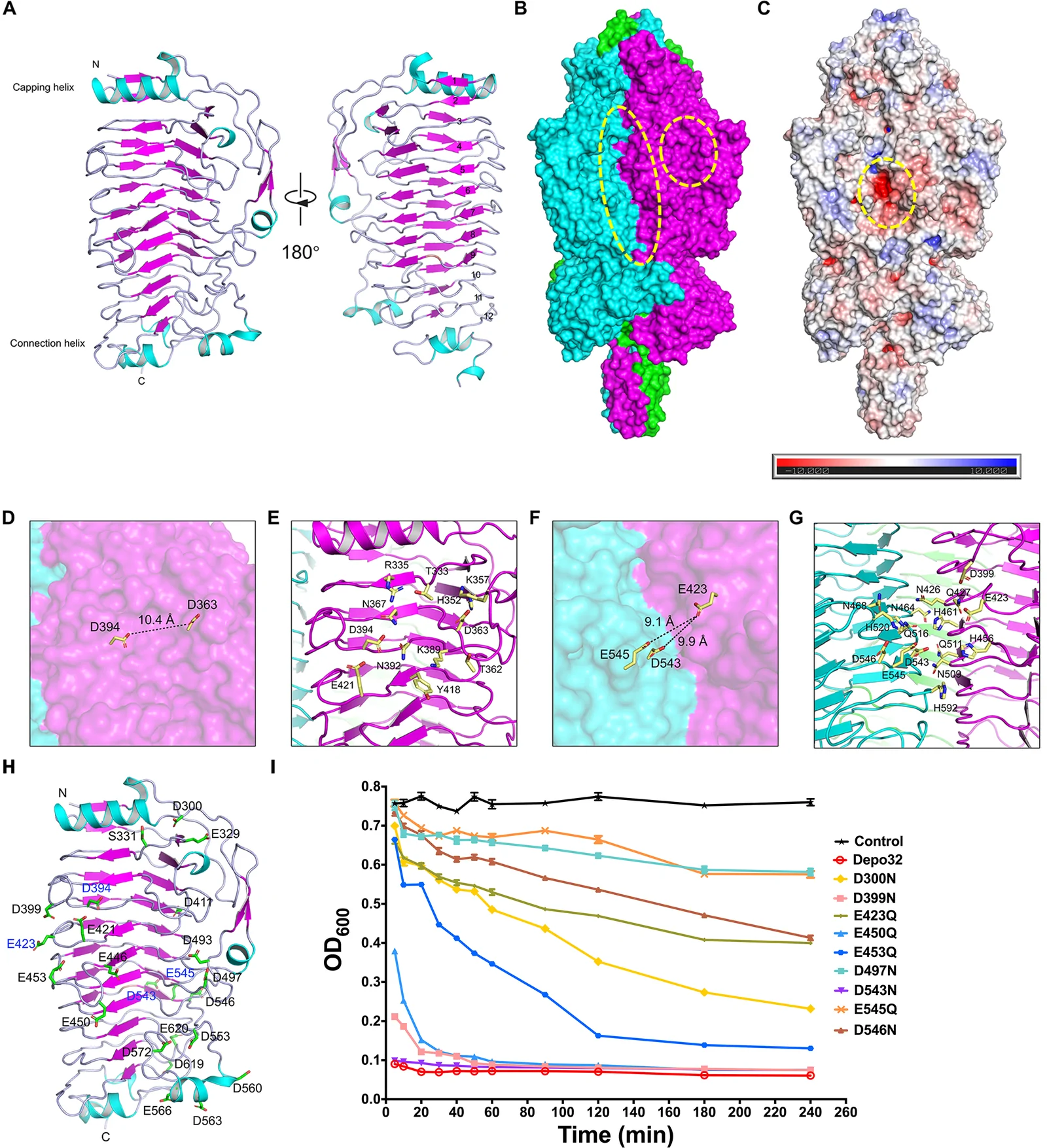
Putative active center and the potential catalytic residues of Depo32 in the β-helix domain. (**A**) Cartoon representation of the β-helix domain of Depo32. There are a total of 12 rungs in the β-sheet ladder; the β-helix domain is initiated from a canonical capping α-helix, and the β-helix domain is terminated by the connection helix. (**B**) Surface representation of the Depo32 trimer. The pockets identified on both the inter- and intrasubunits of the Depo32 trimer are highlighted by yellow ovals. (**C**) Electrostatic surface of the Depo32 trimer. The negatively charged cavity between the adjacent subunits indicating the potential active center is highlighted by the yellow oval. (**D**) The acidic residue pair appeared in the intrasubunit cavity of the β-helix domain. The two residues Asp363 and Asp394 are displayed as sticks, and the distance between the two carboxyl groups is shown by the dashed line. (**E**) Polar residues around the Asp363/Asp394 pair on the concave surface of each β-helix domain; the residues are shown as sticks. (**F**) The acidic residue pairs identified in the intersubunit cavity of the adjacent β-helix domains. The residue pairs Glu545/Glu423 and Asp543/Glu423 are displayed as sticks. The distances between two carboxyl groups are shown by dashed lines. (**G**) Polar residues around the Glu423/Asp543 and Glu423/Glu545 pairs in the groove of the adjacent β-helix domains; the residues are shown as sticks. (**H**) Mutation sites selected on the β-helix domain of Depo32, including the putative Asp or Glu catalytic residues and the other polar residues located far away from the potential substrate binding cleft. (**I**) The mucoviscosity curves of *K. pneumoniae* K7 were recorded within 4 h after Depo32-mutant proteins (Asp300Asn, Asp399Asn, Glu423Gln, Glu450Gln, Glu453Gln, Asp497Asn, Asp543Asn, Glu545Gln, and Asp546Asn) treatment. The OD_600_ of K7 was measured after centrifugation for 5 min at 1,000 × *g* with a starting turbidity of OD_600_ = 1.0. Data represent the mean ± SEM of triplicate experiments.

As observed in a number of phage tail spike proteins (TSPs), the neck helix and the subsequent connection domain of Depo32 form a trimer mainly through hydrophobic interactions and only a few hydrophilic interactions (Fig. S6). As is typical of other TSPs, the β-helix domain of Depo32 adopts a right-handed solenoid-like fold that consists of three β-strands in each coil turn that are stacked approximately in parallel into a triangular cross-section (Fig. 3A; Fig. S5B). The β-helix domain is initiated from a canonical capping α-helix. The linker region that joins the above connection domain and the capping α-helix of the β-helix domain comprises a β-strand that stacks with the first β-strand of the β-helix domain, a turn of the α-helix, and a long loop that is extended from the body of the β-helix domain. As a result, there are a total of 12 rungs in the β-sheet ladder (Fig. 3A). The three β-helix domains from the Depo32 trimer form a left-handed coiled β-coil architecture (Fig. S5B) that is mainly mediated by polar interactions, whereas the residues inside the β-helical solenoid are highly hydrophobic.

After the β-helix domain, the CBM of Depo32 presents a β-sandwich domain that exhibits a “jelly roll” fold, which is composed of eight antiparallel β-strands that are arranged in two layers of four-stranded β-sheets (Fig. S5B and S7A). This domain, despite low sequence identity to the known structures in the database, shares structural similarity to a number of carbohydrate-binding proteins (17 – 19) (Fig. S7B through D).

After the CBM-like domain, the loops extend from the α-helices of the three subunits to generate a three-helix bundle in a clockwise mode through hydrophobic interactions (Fig. S5B). Together with the N-terminal tangled neck helix bundle mentioned above, it seems that Depo32 utilizes the two intertwined ends to cooperatively enhance trimeric assembly, which may ensure and contribute to the stability of the protein as well as the necessity of trimerization for enzymatic activity (18, 20).

### Potential catalytic center in the β-helix domain of Depo32

By analogy to other TSPs, the β-helix domain is the putative polysaccharide binding domain that adheres and lyses the CPS of *K. pneumoniae*. Through comprehensive inspection of the single β-helix domain or the trimer of Depo32, it was observed that both the intra- and intermolecular surface pockets were flanked by pairs of Asp/Glu residues, and the substrate oligosaccharides might associate at this location and be cleaved (Fig. 3B and C). For example, residues Asp363 and Asp394, which localize in the solvent-accessible concave surface of each β-helix domain, were observed. These two residues are 10 Å apart from each other (Fig. 3D and E). In addition, a cluster of negatively charged residues, including Glu545 and Asp543 in one subunit and Glu423 in the adjacent subunit, are located in close proximity within the intermolecular cleft, and surface charge analysis of the molecule showed that these residues are located in a highly negatively charged basin between the neighboring subunits (Fig. 3C). The distances between the carboxyl groups of the Glu545/Glu423 and Asp543/Glu423 pairs were 9.0 ± 0.2 Å and 9.9 ± 0.1 Å, respectively (Fig. 3F). Furthermore, a number of polar residues are observed around the acidic residue pairs, including Thr333, Arg335, His352, Lys357, Thr362, Asn367, Lys389, Asn392, Tyr418, and Glu421 in the concave surface in each monomer as well as Asn464, Asn468, Gln516, His520, and Asp546 from one chain, and Asn426, Gln427, His456, Asn509, Gln511, and His592 from the adjacent subunit; all of these pairs might function in substrate binding as well as stabilizing the reaction intermediate during catalysis (Fig. 3E and G).

To identify the potential active sites of Depo32, all the suspected residues, including the putative Asp or Glu catalytic residues and the other residues located far away from the potential substrate binding cleft of the β-helix domain, were mutated into their isosteric noncharged residues (Fig. 3H; Table 1). The single mutations in residues Asp399 and Asisp546 reduced enzyme activity by 50-fold, while both the mutation of Glu450 and Glu453 caused only a fivefold drop in activity. However, mutations in Glu423, Asp497, and Glu545 all resulted in a near loss of enzymatic activity (Table 1), which indicates that the putative catalytic center is located in the cleft between the neighboring subunits, and it is similar to that of previously identified TSPs (18). In particular, the mucoviscosity of *K. pneumoniae* K7 incubated with Asp497Asn and Glu545Gln showed a limited decrease within 4 h (Fig. 3I). Therefore, residues Glu423, Glu545, and Asp546 constitute the putative active center of Depo32, and Asp497 may also play a key role during catalysis.

**TABLE 1:** The effect of single mutations on the enzyme activity of Depo32^*a*^

Mutation	The lowest enzyme mass with visible activity on bacterial lawn (ng)	Activity reduction compared to wild-type enzyme (fold)
**Depo32 (wild type**)	**1.0**	**1**
D399N	50.0	50
E450Q	5.0	5
E453Q	5.0	5
H671L	25.0	25
D300N	5.0	5
D546N	50.0	50
R810A	5.0	5
E423Q	>2,500	>2,500
D497N	>2,500	>2,500
E545Q	>2,500	>2,500

^*a*^ The following mutant proteins (D300N, E329Q, D394N, D399N, D411N, E421Q, E423Q, E446Q, E450Q, E453Q, D493N, D497N, D543N, E545Q, D546N, D553N, D560N, D563N, E566Q, D572N, D619N, E620Q, Y714A, Y728A, R765A, Q769A, R810A, H671L, N672A, N687A, H690L, R692E, N697A, N707A, R711E, N717A, E735N, E748N, K750E, Q755A, R765E, R799E, K808E, R810E, R824E, N834A, K835E, R836E, K840E, and Y842F) were also expressed and purified, but their enzyme activity was not different from that of Depo32.

### Depo32 promotes macrophage endocytosis and bactericidal activity in human serum

**Fig 4:**
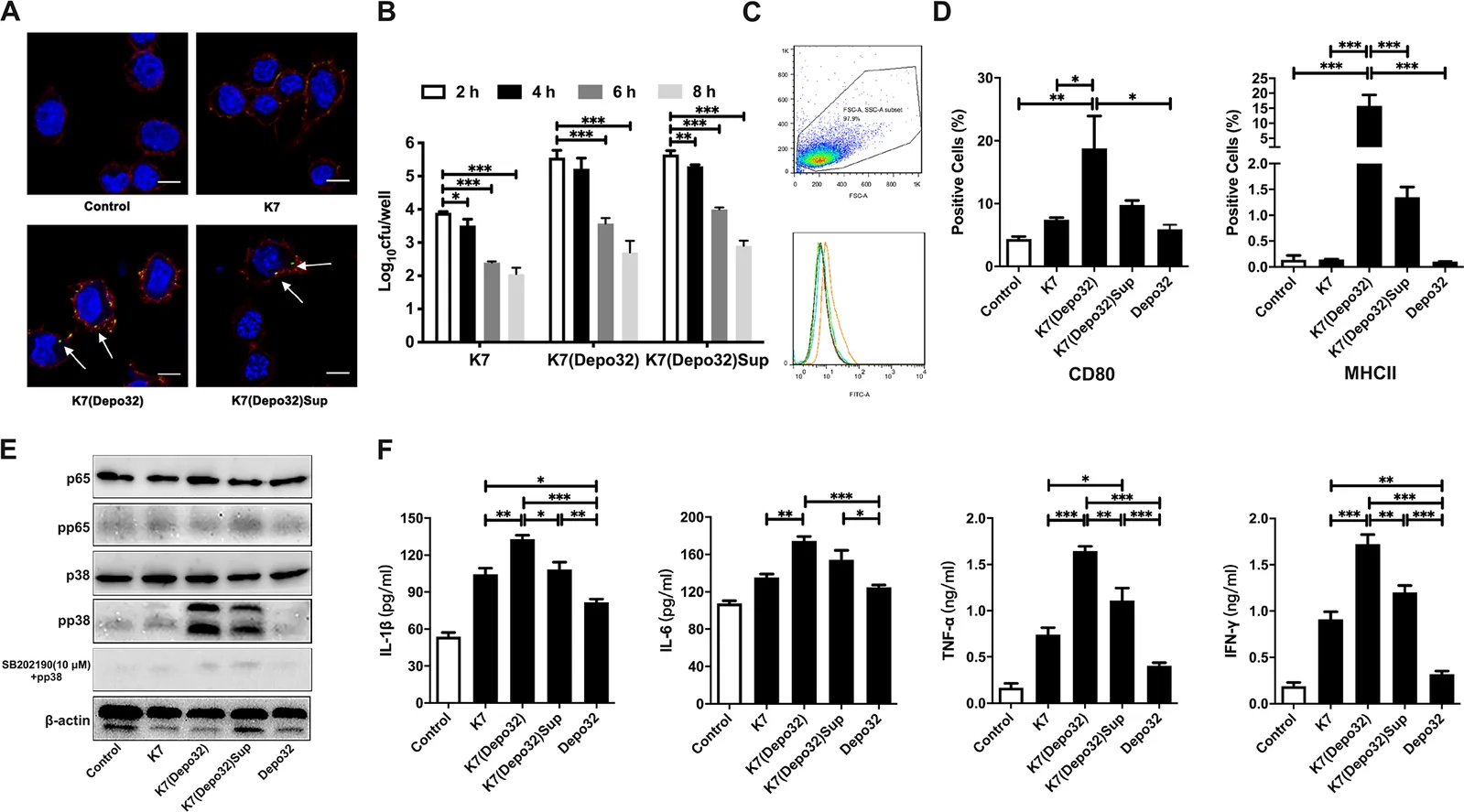
Endocytosis and activation effects of RAW264.7 cells after incubation with different *K. pneumoniae* strains. (**A**) *K. pneumoniae* were stained using SYTO 9 Green Fluorescent Nucleic Acid Stain. *K. pneumoniae* K7 and K7(Depo32) were cocultured with RAW264.7 cells in antibiotic-free supernatants. K7(Depo32)Sup indicates K7 incubated with cells in the presence of 20 µg/mL Depo32. The cytoskeleton and nuclei were stained with Phalloidin-iFluor 555 and Hoechst 33342, respectively. The phagocytosis of *K. pneumoniae* by RAW264.7 cells was detected with a laser scanning confocal microscope at a magnification of 630× (scale bar, 10 µm). (**B**) The bacterial loads of intracellular *K. pneumoniae* in RAW264.7 cells. The cells (5 × 10^5^ cells per well) were incubated with K7, K7(Depo32), or K7 in the presence of Depo32 [K7(Depo32)Sup group] in the supernatant at 37°C for 1 h. After gentamicin treatment for 1, 3, 5, or 7 h and cell lysis, the bacterial loads in each cell sample were determined by plating. (**C**) Example fluorescence-activated cell sorting (FACS) for the measurement of CD80 in macrophages. (**D**) Summary data of CD80 and MHC II expression levels in RAW264.7 cells after incubation. Untreated cells served as controls (*n* = 4). (**E**) Expression levels of p38, p65, pp38, and pp65 in these cells after incubation. SB202190 was used to inhibit p38 MAP kinase. Untreated cells were used as controls. β-Actin served as a loading control. (**F**) After cell-bacteria incubation, the levels of four cytokines (IL-1β, IL-6, TNF-α, and IFN-γ) in the supernatants were detected by ELISA. The untreated cells served as controls. *, **, and *** represent significant differences at *P* < 0.05, *P* < 0.01, and *P* < 0.001, respectively. Data represent the mean ± SEM of triplicate experiments.

K7(Depo32) indicates *K. pneumoniae* K7 pretreated with Depo32 as mentioned previously, and K7(Depo32)Sup indicates *K. pneumoniae* K7 incubated with RAW264.7 cells in the presence of 20 µg/mL Depo32. Within 2h, a small amount of *K. pneumoniae* K7 was taken up by RAW264.7 cells, whereas Depo32 increased the phagocytosis of K7 by nearly 2 log (Fig. 4A and B). Although a large number of bacteria were endocytosed into macrophages under the influence of Depo32, 99.8% of the bacteria were eliminated within 8 h in both the K7(Depo32) and the K7(Depo32)Sup groups (Fig. 4B). However, when mutant proteins Glu423Gln, Glu453Gln, and Asp546Asn were present in the culture medium, the endocytosed bacteria showed a significant decrease compared to the K7(Depo32)Sup group (Fig. S8).

The presence of K7(Depo32) significantly induced the expression of specific marker molecules on macrophage surfaces, such as CD80 (12.9–34.3%) and MHC II (8.56–19.9%) (Fig. 4C and D). K7 or Depo32 alone is not effective in activating cellular signaling pathways. However, RAW264.7 cells in both the K7(Depo32) and K7(Depo32)Sup groups showed a sharp increase in the expression levels of phospho-p38 (pp38), especially in the former. However, even when cocultured with K7(Depo32), the expression level of phospho-p65 (pp65) in macrophages remained unchanged (Fig. 4E). This finding suggests that macrophage activation by Depo32-treated *K. pneumoniae* relies on the mitogen-activated protein kinase (MAPK) signaling pathway rather than NF-κB. Simultaneously, the increased accumulation of proinflammatory cytokines, such as IL-1β, IL-6, TNF-α, and IFN-γ, was also detected in the cell supernatants from cells that were cocultured with K7(Depo32) (Fig. 4F). Taken together, these results indicate that the activation of macrophages by *K. pneumoniae* K7 is greatly enhanced after treatment with Depo32.

The killing effect of serum on Depo32-treated *K. pneumoniae* K7 was also evaluated. Normal human serum inhibited the growth of enzyme-treated bacteria within 5 h, and this growth inhibition became more pronounced with increasing doses of the enzyme (2–200 µg/mL) (Fig. S9). However, heat-inactivated human serum had no such effect. Therefore, Depo32 enhances the sensitivity of *K. pneumoniae* to serum killing.

### Depo32 exhibits protective effects against *K. pneumoniae* infection in a mouse model

**Fig 5:**
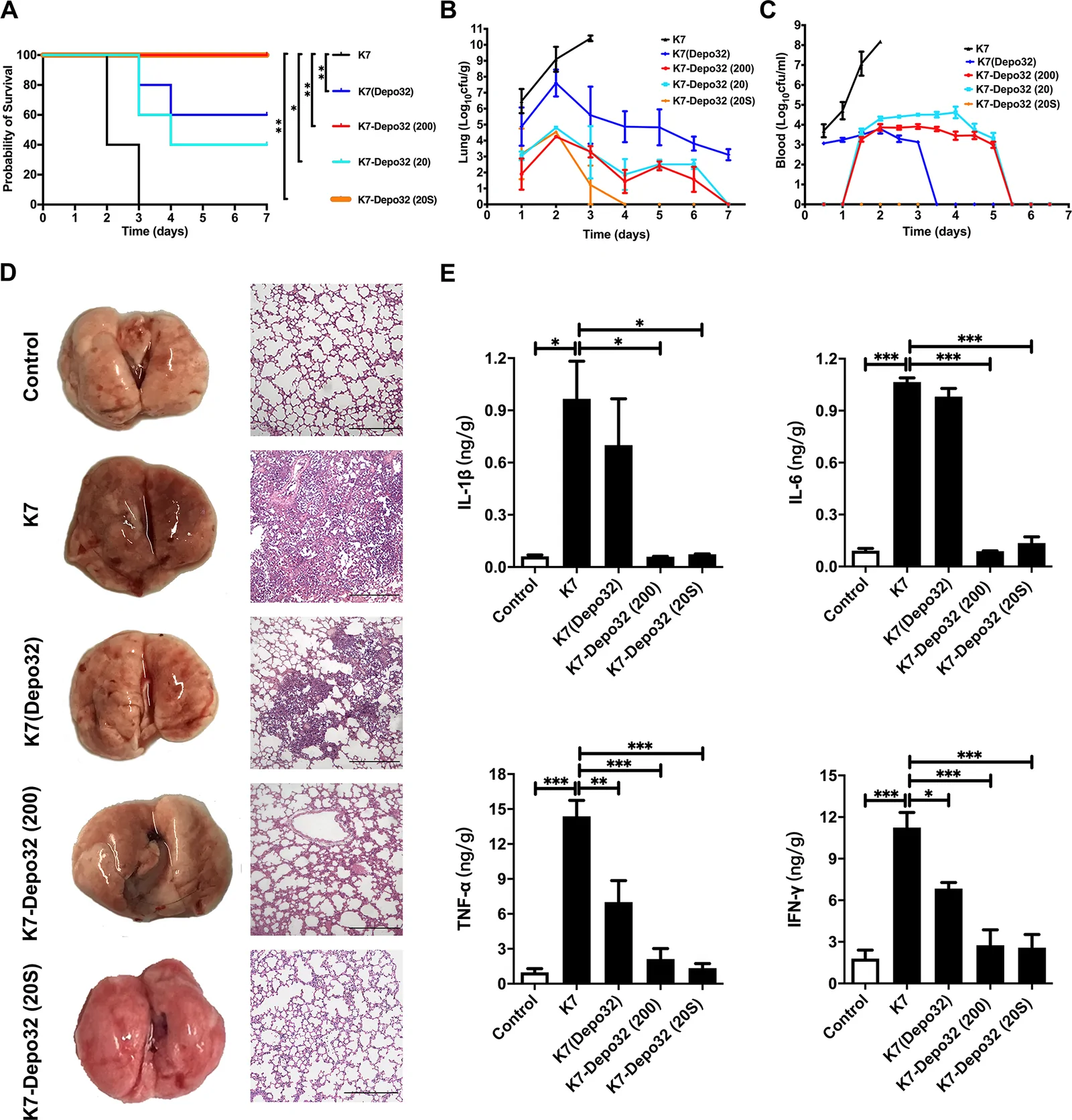
Therapeutic effects of Depo32 on *K. pneumoniae in vivo*. Mice were challenged intranasally with 1.0 × 10^7^ CFU/mouse *K*. *pneumoniae* K7 or K7(Depo32). At 1 h after K7 challenge, mice were treated with 200 µg [K7-Depo32 (200) group] or 20 µg [K7-Depo32 (20) group] of Depo32 in a single dose or 20 µg of protein daily for 3 days [K7-Depo32 (20S) group]. As a negative control, one group was treated with Tris buffer following the K7 challenge. (**A**) Survival rate. The survival rates of mice in the K7, K7(Depo32), K7-Depo32 (200), K7-Depo32 (20), and K7-Depo32 (20S) groups were determined (*n* = 5). The Kaplan‒Meier method [*P* < 0.0001, log-rank (Mantel‒Cox) test] was used for statistical analysis. Dynamic changes in bacterial loads in the (**B**) lungs and (**C**) blood of mice in the K7, K7(Depo32), K7-Depo32 (200), K7-Depo32 (20), and K7-Depo32 (20S) groups. Each symbol represents the average of the results of three experiments (*n* = 3). (**D**) At 48 h after infection, the lungs of mice in the K7, K7(Depo32), K7-Depo32 (200), and K7-Depo32 (20S) groups were carefully removed and photographed after euthanasia. Lung tissue sections were stained with hematoxylin and eosin (scale bar, 100 µm). (**E**) The levels of four cytokines (IL-1β, IL-6, TNF-α, and IFN-γ) in the lung tissues of mice in the K7, K7(Depo32), K7-Depo32 (200), and K7-Depo32 (20S) groups. *, **, and *** represent significant differences at *P* < 0.05, *P* < 0.01, and *P* < 0.001, respectively. Data represent the mean ± SEM of triplicate experiments.

All C57BL/6J mice were intranasally administered the same dose of *K. pneumoniae* strains (1.0 × 10^7^ CFU). The mice in the K7-challenged group all died within 72 h. In contrast, the mortality rate reached 20% at 72 h and 40% at 7 days post-challenge with K7(Depo32), the Depo32-pretreated K7 (Fig. 5A). However, the survival rate of mice challenged with K7 pretreated with Depo32-mutant proteins (Glu423Gln, Asp497Asn, Glu545Gln, and Asp546Asn) was no more than 30% (Fig. S10A). In the K7-challenged group, the bacterial load in the lungs and blood reached 5.1 × 10^9^ CFU/g and 1.6 × 10^8^ CFU/mL at 48 h after infection, respectively. At the same timepoint, the load of K7(Depo32) in the lungs and blood reached approximately 6.1 × 10^8^ CFU/g and 8.3 × 10^3^ CFU/mL, respectively. K7(Depo32) disappeared in the blood within 4 days, but the bacteria in the lungs remained for 7 days after infection (Fig. 5B and C). Additionally, the lung tissues of these moribund K7-group mice were swollen and crimson in color. The histopathological results showed that alveolar structures disappeared quickly, and many inflammatory cells had infiltrated into the tissues. However, the lung tissues of the mice infected with K7(Depo32) were far less hyperemic, and the local pathological changes became weaker (Fig. 5D). Moreover, K7 infection significantly induced the expression of proinflammatory cytokines, but the levels of TNF-α and IFN-γ were significantly lower in the K7(Depo32)-treated group (Fig. 5E).

Mice were intranasally treated with 200 µg [K7-Depo32 (200) groups] or 20 µg [K7-Depo32 (20) groups] of Depo32 (suspended in 50 µL of Tris buffer) at 1, 2, or 12 h after K7 challenge. At 1 h post-K7 infection, the K7-Depo32 (200) group achieved 100% protection after a single dose of 200 µg of Depo32 administration (Fig. 5A). The group showed only mild pulmonary congestion. Despite incurring partial collapse of the alveolar wall and the presence of telangiectasia, most of the alveolar structures were still intact (Fig. 5D). However, when the same dose of Depo32 administration was delayed for more than 2 h after K7 infection, the protective effect of the depolymerase was dramatically reduced (Fig. S10B). Only 40% of the mice were protected by a single dose of 20 µg of Depo32 after K7 challenge for 1 h, with longer delays resulting in worse protection rates (Fig. 5A; Fig. S10B). When treated continuously with 20 µg of Depo32, the exciting thing was that all the mice in K7-Depo32 (20S) group were rescued after 3 days of daily administration (Fig. 5A). Interestingly, the bacteria in the lungs of the K7-Depo32 (20S) mice were completely eliminated within 4 days, and no bacteria were detected in the blood (Fig. 5B and C). Both the lung pathology and cytokine levels in the K7-Depo32 (20S) group were close to those of healthy mice (Fig. 5D and E). In addition, all mice infected with K7 survived after combination therapy with 20 µg of Depo32 and gentamicin (1.5 mg/kg of body weight), which was much higher than the survival rate of mice treated with the same dose of depolymerase or gentamicin alone (Fig. 5A; Fig. S10C). These results suggest that Depo32 has potential not only as an antivirulence agent but also in combination with antibiotics against *K. pneumoniae* infection.

**Fig 6:**
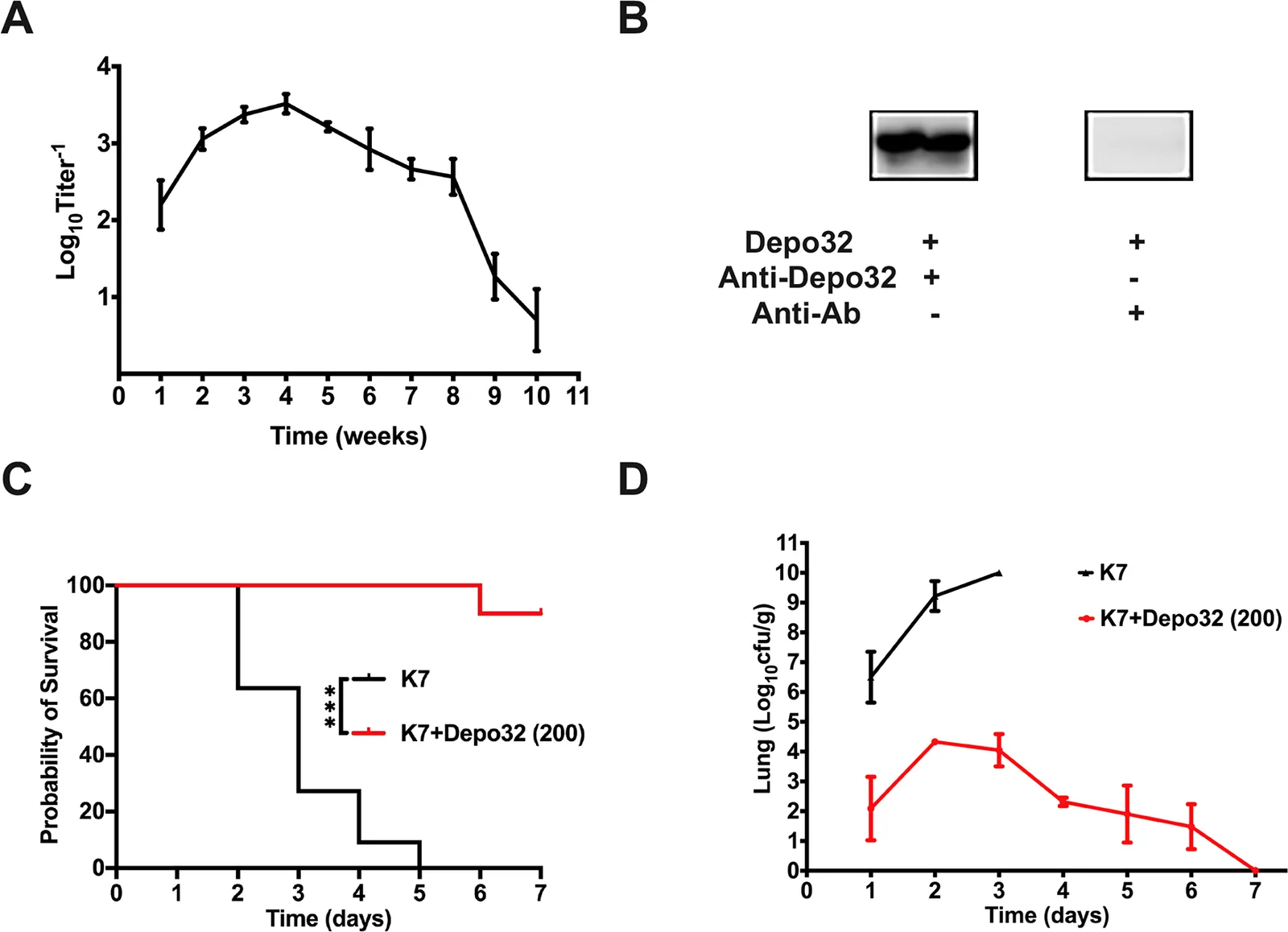
Evaluation of neutralizing antibody on the therapeutic effects of Depo32. (**A**) Within 10 weeks, the change in antibody titers in Depo32-immunized mice was detected by ELISA. (**B**) Sera with a peak titer of Depo32-neutralizing antibody were diluted (1:500) and detected by immunoblotting. HRP-labeled anti-mouse IgG antibody (ab205719, Abcam, MA, USA) was used as the secondary antibody (1:5,000). (Left) sera from Depo32-injected mice and (right) sera from Tris buffer-injected mice served as controls. (**C**) Mice with a peak concentration of Depo32-neutralizing antibody were treated with 200 µg of Depo32 [K7 + Depo32 (200) group] at 1 h after *K. pneumoniae* K7 challenge. The survival rate was then recorded within 7 days (*n* = 10). (**D**) The bacterial loads in the lung tissues of the K7 + Depo32 (200) group. Each symbol represents the average of the results of three experiments (*n* = 3). The K7-infected group that was treated with Tris buffer served as a control.

The level of Depo32-neutralizing antibody in the mice peaked in the fourth week after subcutaneous immunization (Fig. 6A), with good reactogenicity with Depo32 (Fig. 6B). The therapeutic effect of 200 µg of Depo32 on K7 infection in mice at this stage was slightly lower than that of nonimmunized mice (survival rate: 90% vs 100%) (Fig. 6C). The efficiency in clearing lung bacteria in the first 4 days was slightly inferior to that of nonimmunized mice, but with Depo32 treatment, the lung pathogens were eliminated within 7 days (Fig. 5B; Fig. 6D). These data indicate that neutralizing antibody does not appear to affect the therapeutic effect of Depo32 against *K. pneumoniae* infection.

## DISCUSSION

Similar to other phage depolymerases, Depo32 derived from phage GH-K3 was effective in hydrolyzing CPSs on the surface of K2 serotype *K. pneumoniae*. During the preparation of our manuscript, the cryo-EM structure of a Depo32-like depolymerase was reported at a 2.7 Å resolution (21). Since the acidic side chains are highly sensitive to radiation damage from electron irradiation (22, 23), our structures solved at much higher resolution offer more accurate models as well as unambiguous side-chain densities for negatively charged residues, which facilitates the interpretation of the catalytic mechanism. By dissecting the cryo-EM structure of the Depo32 trimer at 2.32 Å resolution, we identified two acidic residue pairs formed by Glu545/Glu423 and Asp546/Glu423, and these pairs were predicted to be the putative active sites of the protein. Furthermore, mutating any site into an isosteric noncharged residue led to a severe decrease or even loss of enzyme activity. The glycosidase catalytic mechanism of hydrolases generally relies on the formation of negatively charged cavities by two adjacent Asp/Glu amino acid residues, in which the carboxyl groups are involved in the acid/base mechanism that is necessary for the hydrolysis of target substrates (18, 24), and Depo32 clearly has these features. The distance between the two carboxyl groups is usually between 5 and 11 Å; therefore, two modes of action are proposed for glycosidases, i.e., the inverting mechanism and the retaining mechanism. The two carboxyl groups that are located 10.5 Å apart on average in inverting glycosidases accommodate the substrate and a water molecule that serves as the nucleophile to bind between them, and the hydrolysis reaction occurs through a single-displacement mechanism that involves an oxocarbenium ion-like intermediate. This mechanism results in inversion of stereochemical configuration at the anomeric carbon. In contrast, in the retaining glycosidases, the two carboxyl groups are positioned approximately 5.5 Å apart, and the glycosidic bonds break via two sequential steps that involve a covalent glycosyl-enzyme intermediate; this is consistent with a double-displacement mechanism and leads to the preservation of the stereochemical configuration at the anomeric carbon. The mechanism of the carboxyl group action in *Klebsiella* phage depolymerases has rarely been reported. Previously, only KP32gp38 was shown to have a retaining acid/base mechanism (18). However, our data supported that Depo32 has an inverting catalytic mechanism.

In addition to causing the degradation of CPSs, phage depolymerase cannot affect the vitality of bacteria. However, the depolymerase-treated strains lost their capsule and tended to be bound by complement C3, greatly increasing their probability of undergoing phagocytosis by macrophages (1). The phagocytized bacteria can eventually be cleared by neutrophil or complement-mediated cell-killing effects (25, 26). In the present work, the Depo32-treated strains were more susceptible to recognition and killing by immune cells than the nontreated strains, suggesting that the unencapsulated strains might also trigger complement-mediated opsonophagocytosis. The survival rate of the mice challenged with K7(Depo32) lagged behind that of the K7-Depo32(200) group (1 h post-infection) in our study (60% vs 100%). Our further study found that the capsules of K7(Depo32) were regenerated after subculture in the absence of depolymerase (data not shown). These results may indicate that progeny bacteria restored the biosynthesis of CPSs after the depolymerase pressure was released, thus preventing macrophage recognition and phagocytosis. It is worth noting that Depo32 has a stable therapeutic effect in the presence of neutralizing antibody, and its enzymatic activity is almost unaffected by the neutralizing effect of antibody *in vitro* (Fig. S11A). This outstanding feature enhances the application potential of this depolymerase. However, the Depo32-neutralizing antibody effectively blocked the infection of phage GH-K3 in a dose-dependent manner (Fig. S11B), indicating that this antibody can block the host receptor binding domain of the depolymerase.

Although depolymerases are effective in controlling bacterial infections, their therapeutic effect is usually decreased when the treatment is delayed (25). The present study also found that both 200 µg and 20 µg of Depo32 exhibited lower protective rates after delayed treatment (2 or 12 h). However, to our great satisfaction, the daily administration of 20 µg of Depo32 for 3 days rescued all the mice infected with a lethal dose of K7 and quickly eliminated the bacteria from the lungs without the development of bacteremia. A previous study indicated that *K. pneumoniae* treated with the phage-derived depolymerase Dep42 has increased sensitivity to polymyxin (27). Additionally, the phage depolymerase Dpo71 that specifically targets multidrug-resistant *Acinetobacter baumannii* has exhibited effective potential for treatment in a *G. mellonella* model when combined with colistin (28), demonstrating that depolymerases have an excellent synergistic effect with antibiotics. Our study showed that Depo32 in combination with gentamicin rescued 100% of K7-infected mice, which was more effective than treatment with Depo32 or gentamicin alone. Thus, when the efficacy of depolymerase treatment is limited, continuous administration of depolymerase or combination therapy with antibiotics may be a better choice.

In summary, Depo32 effectively binds and cleaves the CPSs of K2 capsular type *K. pneumoniae* via the putative active center constituted by Glu545/Glu423 and Asp546/Glu423 and initiates the hydrolysis process, causing the pathogens to become sensitive to elimination by the host immune system and thus achieving protective effects. Depo32 not only shows potential as a candidate antivirulence agent against the K2 serotype *K. pneumoniae* but also the antimicrobial mechanism of the depolymerase has also been elucidated. Due to the rapid development of enzyme engineering technology, it is possible to construct or modify depolymerases to target CPSs with specific serotypes (15). Therefore, further exploration of the construction of serotype-broad enzymes to combat complex *K. pneumoniae* infections based on the structural biological data of Depo32 is a worthwhile pursuit.

## MATERIALS AND METHODS

### Bacterial strains and culture conditions

*K. pneumoniae* K7 (29, 30) and other *Klebsiella* strains were isolated from clinical specimens and maintained in our laboratory. The capsular types of these strains were identified according to the description in a previous study (31). The strains were routinely cultured in Luria-Bertani (LB) broth at 37°C with shaking at 180 rpm. Procedures for capsule staining, scanning electron microscopy, and CPS analysis are detailed in the supplemental materials.

### Protein expression and purification

Based on the genomic DNA of phage GH-K3, the *depo32* (*gp32*) gene was amplified using the following primers: F, CGCCATATGATGGCACTATACAGAGAAGGCA and R, CCGCTCGAGTTAGCTACTCATAAATCCATTTGC. The PCR product was ligated into the pET28a(+) vector (Novagen, Madison, WI, USA) and subsequently validated by sequencing. Using pET28a-*depo32* as a template, mutation primers (Table S2) were designed according to the predicted mutation sites (Table 1) and then amplified by a GeneArt Site-Directed Mutagenesis PLUS System (Invitrogen, Carlsbad, CA, USA). The plasmids were then transformed into *E. coli* BL21 (DE3) (TransGen Biotech, Beijing, China). The overnight BL21 cultures were inoculated into LB broth (50 µg/mL kanamycin) and cultured for 2–3 h until the exponential phase (OD_600_ 0.6–0.8) was reached. Isopropyl-β-D-thiogalactopyranoside was then added (1 mM) and cultured with shaking at 16°C for 16 h. Depo32 and site-directed mutagenesis proteins were purified as previously described (32). Briefly, cell pellets were harvested by centrifugation (6,000 × *g*, 10 min, and 4°C), washed three times and resuspended with prechilled Tris buffer (20 mM Tris, pH 8.0; and 200 mM NaCl), and lysed by ultrasonication on ice. The insoluble cell debris was removed by centrifugation at 21,000 × *g* for 30 min at 4°C. The supernatants were then gently transferred and incubated with equilibrated nickel-nitrilotriacetic acid resin, washed through a gravity column (GE Healthcare, Uppsala, Sweden) using Tris buffer supplemented with 20 and 50 mM imidazole sequentially, and finally eluted with 300 mM imidazole. For further purification, the eluted proteins were concentrated and applied to a Superdex 200 Increase 10/300 GL gel filtration column (Cytiva, Marlborough, MA, USA). The fractions eluted from the gel filtration column were pooled, concentrated, quantified by A280, frozen in liquid nitrogen, and stored at −80°C until use. Procedures for the effect of neutralizing antibody on this protein are detailed in the supplemental materials.

### Stability analysis of Depo32

The concentration of Depo32 was adjusted to 100 µg/mL. To determine the influence of diverse pH values on the enzyme activity, Depo32 was treated with 50 mM sodium acetate (pH 3.0–6.0) or 20 mM PBS (pH 7.0–11.0) for 1 h. To evaluate the effect of temperature on the enzyme activity, assays were performed in 20 mM PBS (pH 7.0) in the range of 4–80°C for 1 h. To detect the dependence of Depo32 on metal ions and the impact of high salinity on enzyme activity, the depolymerase was processed using EDTA (1 mM EDTA; 20 mM Tris, pH 8.0; and 0.2 M NaCl) and NaCl (5 M), respectively, followed by buffer exchange through ultrafiltration. The enzyme activity of the Depo32 that was subjected to different treatments was immediately detected by spot assays. Briefly, 5 µL of 10-fold serial diluted depolymerase solutions were continuously spotted onto freshly seeded lawns of *K. pneumoniae* and were incubated for 6 h at 37°C before observation.

### Cryo-EM sample preparation and data collection

For the cryo-EM sample preparation, 3 µL of size exclusion chromatography purified Depo32 at a concentration of 0.5–1.0 mg/mL was loaded onto a glow-discharged C-flat carbon grid (1.2/1.3, 300 mesh). The grids were blotted for 3.5 or 4.0 s with 100% humidity at 12°C and were plunge-frozen into liquid ethane using Vitrobot Mark IV. The grids were then screened by a 200 kV Talos Arctica transmission electron microscope, and data acquisition was performed on a 300-kV Titan Krios transmission electron microscope (all equipment from FEI Inc., Hillsboro, OR, USA) at the Cryo-EM center of Westlake University; all of these were equipped with a K3 direct electron detector (Gatan Inc., Pleasanton, CA, USA). Specifically, movies were collected at a nominal magnification of 81,000×, corresponding to a calibrated pixel size of 1.087 Å on the specimen level and 0.5435 Å to obtain a super-resolution. The dose rate was set to 23 counts per physical pixel per second. The total exposure time for each movie was 2.56 s (80 ms per frame), resulting in a total accumulated dose of 50 electrons per Å^2^, fractionated into 32 frames (1.56 electrons per frame). All movies were recorded using a defocus ranging from −1.5 to −2.0 µm.

### Cryo-EM data processing and 3D reconstruction

The dose-fractionated movies that were acquired by the superresolution mode were binned over 2 × 2 pixels, yielding a pixel size of 1.087 Å, and were then subjected to drift correction, beam-induced motion, and dose-weighting by the program MotionCor2 (33), resulting in a series of corrected and summed micrographs, including dose-weighted and nondose-weighted micrographs. Contrast transfer function (CTF) parameters were estimated by GCTF (a robust Graphic Processing Unit (GPU)-accelerated computer program for CTF determination, refinement and correction) implanted in RELION 3.1.0 (34) using nondose-weighted micrographs. Approximately 5,000 particles were boxed manually from the dose-weighted micrographs and then extracted and subjected to successive rounds of 2D classification within RELION (34). Subsequently, the templates that represented the different distributions of projections of the particles were selected as the templates for particle autopicking for all the micrographs. A total of 1,767,920 particles were picked, and a box size of 256 pixels was used to extract the particles. The autopicked particles were further cleaned through multiple rounds of 2D classification, and 1,084,534 particles were selected to perform the 3D reconstruction in RELION (34). The initial model that was necessary for 3D classification was generated using a small number of particles from each 2D class average through the 3D initial model operation in RELION (34). The orientation parameters of the homogenous set of particle images in the selected 3D classes that contained 462,388 particles were iteratively refined to yield higher resolution maps using the “autorefine” procedure. C3 symmetry was also imposed during the refinement process to obtain a higher resolution for the map in the symmetrical region according to the symmetry of the molecule itself. All reported resolutions were based on gold-standard refinement procedures and the Fourier shell correlation = 0.143 criterion. Local resolution was estimated using ResMap (35) by the two half maps (Table S1).

### Model building and refinement

The nonsymmetried 2.4 Å-resolution map and C3-symmetrized 2.3 Å-resolution map were used for building the models. The initial model was obtained through the map-to-model program within PHENIX (36) using the higher-resolution C3-symmetrized map. Then, the main and side chains were manually adjusted or rebuilt residue by residues in COOT (37). To obtain better side-chain densities for model building, different B-factors were varied and applied for map sharpening, and this was performed according to the values of auto-B-factors that were reported from the RELION postprocess jobs (34). Then, the model built from the higher-resolution map was positioned into the nonsymmetrized map through translation and rotation using UCSF Chimera (38), and the model was further completed in COOT (37). The maps were placed into an artificial unit cell with P1 symmetry and converted to MTZ format using phenix.map_to_structure_factors (36). The resulting reflection files were used to iteratively perform refinements in real and reciprocal spaces in PHENIX (36) with secondary structure restraints, X-ray and stereochemistry weight restraints, rotamer restraints, and Ramachandran plot restraints. The geometries of the final models were validated using Molprobity (39). All molecular representations were generated in PyMOL (http://www.pymol.org) [Delano, W.L. The PyMol Molecular Graphics System (2002)] and UCSF Chimera (38). DALI was used to perform protein structure comparisons (40).

### Macrophage phagocytosis and activation

Macrophage phagocytosis and activation induced by *K. pneumoniae* were measured at a multiplicity of infection of 10:1. The maintenance of the RAW264.7 cell line (ATCC HTB37) and the immunofluorescence assay were performed according to our previously described methods (41). Briefly, *K. pneumoniae* K7 and K7(Depo32) were stained using SYTO 9 Green Fluorescent Nucleic Acid Stain (catalog number: S34854; Invitrogen, Carlsbad, CA, USA). RAW264.7 cells were incubated with *K. pneumoniae* K7, K7(Depo32), and K7 in the presence of 20 µg/mL Depo32 [K7(Depo32)Sup group]. After 2 h of incubation, the cytoskeleton and nuclei of cells were identified by incubation with Phalloidin-iFluor 555 (ab176756; Abcam, MA, USA) (1:200) and Hoechst 33342 (Invitrogen, Waltham, MA, USA) (1:200), respectively. Endocytosis of the cells that were treated with different *K. pneumoniae* strains was then determined using laser scanning confocal microscopy (LSM710; Carl Zeiss, Germany). Additionally, after incubation with *K. pneumoniae* strains at 37°C for 1 h, the cells (5 × 10^5^ cells per well) from the K7, K7(Depo32), and K7(Depo32)Sup groups were treated with gentamicin for 1, 3, 5, or 7 h and lysed, and the intracellular bacteria were quantified by plating based on a previously described method (42).

Activated macrophages usually express specific marker molecules, such as CD80, a costimulatory molecule, and MHC II, an efficient antigen-presenting molecule (43). RAW264.7 cells were incubated with *K. pneumoniae* K7, K7(Depo32), K7(Depo32)Sup, or Depo32 for 2 h. To detect these specific marker molecules on the cell surface, the cells were labeled with FITC-conjugated anti-mouse CD80 antibody (553768; BD Biosciences, USA) and PE-conjugated anti-mouse MHC II antibody (12–5322; eBioscience, USA). The cells were then analyzed by an LSRFortessa analyzer cytometer (BD Biosciences, Franklin Lakes, NJ, USA). MAPK and NF-κB signaling pathways are also involved in macrophage activation (43). Total cellular proteins from the K7, K7(Depo32), K7(Depo32)Sup, and Depo32 groups were extracted and quantified, and the expression levels of p38, p65, pp38, and pp65 in the cells were examined by immunoblotting as previously described (41). Cytokine expression levels in the culture supernatants, including IL-1β, IL-6, TNF-α, and IFN-γ, were detected by enzyme-linked immunosorbent assay (ELISA) kits (eBioscience, San Diego, CA, USA).

The procedures for the serum sensitivity assay of Depo32-treated *K. pneumoniae* are detailed in the supplemental materials.

### Therapeutic effects of Depo32 against *K. pneumoniae in vivo*

The protective effects of Depo32 on the mouse pneumonia model were determined, and the grouping information is shown in Table S3. With a minimum lethal dose (MLD) of 5 × 10^6^ CFU/mouse (41), *K. pneumoniae* K7 was used to establish a mouse model of acute pneumonia. Mice were anesthetized intraperitoneally with ketamine (100 mg/kg of body weight) and xylazine (10 mg/kg of body weight) and were challenged intranasally with 2 × MLD (1 × 10^7^ CFU) of K7 or the same dose of other *K. pneumoniae* strains. Six groups of K7-challenged mice were then intranasally treated with 200 µg [K7-Depo32 (200) group] or 20 µg [K7-Depo32 (20) group] of Depo32 at 1, 2, or 12 h post-infection once, and one group was treated with 20 µg of protein daily for three consecutive days [K7-Depo32 (20S) group]. To compare the therapeutic effects of Depo32 and gentamicin on *K. pneumoniae* infection, one group of K7-challenged mice was intranasally treated with gentamicin (1.5 mg/kg of body weight) at 1 h of infection. To evaluate the therapeutic effect of Depo32 combined with gentamicin, another group of K7-challenged mice was intranasally treated with 20 µg of Depo32 and gentamicin (1.5 mg/kg of body weight) at 1 and 2 h of infection, respectively. The control group was treated with Tris buffer following K7 challenge. The survival rate of each group was recorded every day for 7 days.

Four groups of mice were challenged with K7 (1 × 10^7^ CFU). At 1 h after the challenge, three groups were treated with 200 µg [K7-Depo32 (200) group] or 20 µg [K7-Depo32 (20) group] of Depo32 or Tris buffer (K7 group). The remaining K7-infected group was treated with 20 µg protein daily for 3 days [K7-Depo32 (20S) group]. Additionally, a group of mice was infected with the same dose of K7(Depo32). Three mice were randomly selected from each group every 24 h and euthanized with an intravenous injection of Fatal Plus (sodium pentobarbital) (100 mg/kg of body weight) for 7 days after infection. Bacterial loads, histopathological damage, and cytokine expression were determined as previously described (41, 44).

To test the effect of neutralizing antibody on the therapeutic efficacy, 50 µg of Depo32 was administered subcutaneously to mice. Serum samples were then collected weekly (for a total of 10 weeks), and antibody titers were detected by ELISA. Mice with peak concentrations of neutralizing antibody were infected with *K. pneumoniae* K7 (1 × 10^7^ CFU) and treated with Depo32 (200 µg, 1 h post infection). The survival rate of these mice and bacterial loads in the lung tissues (*n* = 3 per day) were then recorded within 7 days. All the experiments reached the endpoint 7 days postinfection, and the surviving mice were euthanized as described above.

### Statistical analysis

Survival curve analysis was performed using the log-rank (Mantel–Cox) test, and other statistical data were processed by one-way analysis of variance or Student’s *t*-tests. All charts were generated by GraphPad Prism 9 (GraphPad Software, Inc., CA, USA). *P* values < 0.05 were considered statistically significant. Error bars represent the standard error of the mean.

## Data Availability

The atomic coordinates and cryo-EM maps are available in the PDB and EMDB databases under the accession numbers PDB 7VZ3 and 7VYV for non- and C3-symmetrized models, respectively, and EMD-32219 and EMD-32215 for non- and C3-symmetrized maps, respectively. The data sets generated during the current study are available from the corresponding authors upon reasonable request.

## SUPPLEMENTAL MATERIAL

Supplemental material spectrum.05304-22-s0001.pdf Fig. S1 to S11, Tables S1 to S3.

ASM does not own the copyrights to Supplemental Material that may be linked to, or accessed through, an article. The authors have granted ASM a non-exclusive, world-wide license to publish the Supplemental Material files. Please contact the corresponding author directly for reuse.
