# Supplementary material for: Structural biology and functional features of phage-derived depolymerase Depo32 on Klebsiella pneumoniae with K2 serotype capsular polysaccharides
Source: Microbiol Spectr. 2023 Sep 26;11(5):e05304-22. doi: 10.1128/spectrum.05304-22 (PMC10581125; doi:10.1128/spectrum.05304-22)
Supplement: Supplemental material — Fig. S1 to S11, Tables S1 to S3. [file spectrum.05304-22-s0001.pdf]

Supplementary Materials

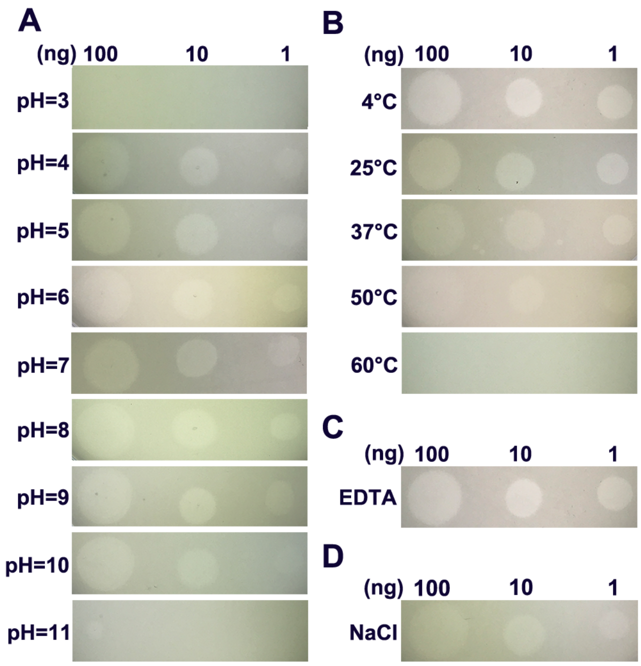

FIG S1 Enzyme stability of Depo32. Depo32 (100 µg/ml) solutions were maintained at (A) various pH values (pH 3-11, 25 °C), (B) temperatures (4-80 °C), (C) high concentrations of EDTA (0.25 M), and (D) NaCl (5 M) conditions for 1 h. Depo32-treated solutions were diluted and spotted onto *K. pneumoniae* K7 lawns (1 ng, 10 ng, and 100 ng).

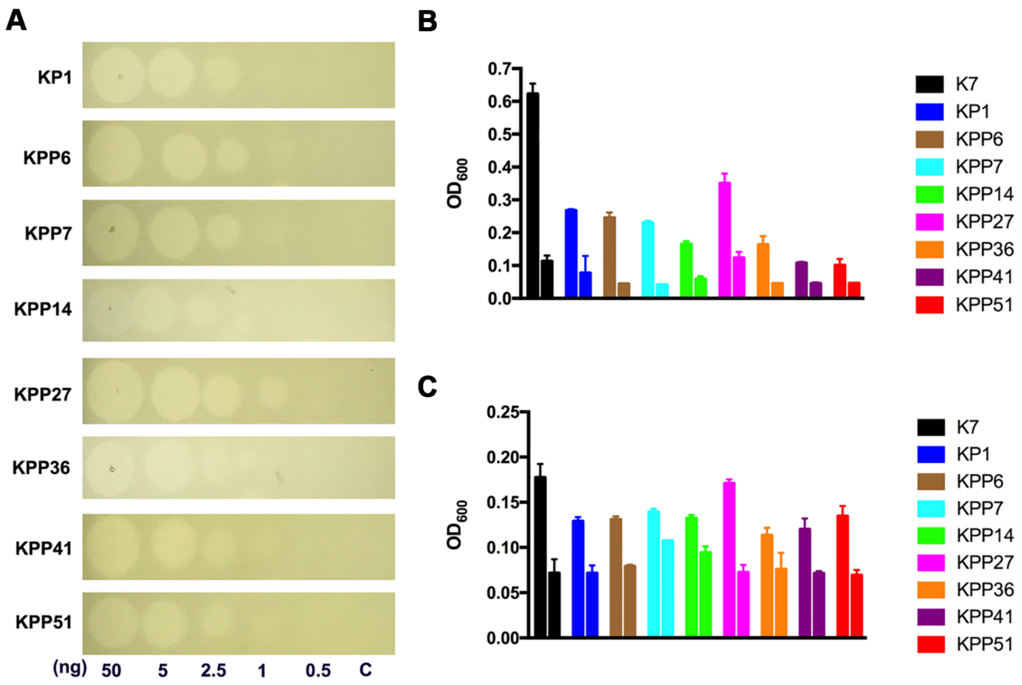

FIG S2 CPS degradation effect of Depo32 on K2 serotype *K. pneumoniae*. (A) Enzyme activity assay. Depo32 (100 µg/ml) solutions were serially diluted and spotted on the lawns of K2 serotype *K. pneumoniae*, including KP1, KPP6, KPP7, KPP14, KPP27, KPP36, KPP41 and KPP51 (0.5-50 ng). Tris buffer was used as a control. (B) Mucoviscosity. Before (left columns) or after (right columns) Depo32 treatment, the OD_{600} of K7 and other *K. pneumoniae* strains was measured after centrifugation for 5 min at 1,000 g with a starting turbidity of $OD_{600} = 1.0$. Data represent the mean ± SEM of triplicate experiments. (C) Before (left columns) or after (right columns) Depo32 treatment, CPSs derived from K7 and other *K. pneumoniae* strains were precipitated by adding cetylpyridinium chloride (CPC) to a final concentration of 5 mg/mL. After 10 min of incubation at 25 °C, the OD_{600} was measured. Data represent the mean ± SEM of triplicate experiments.

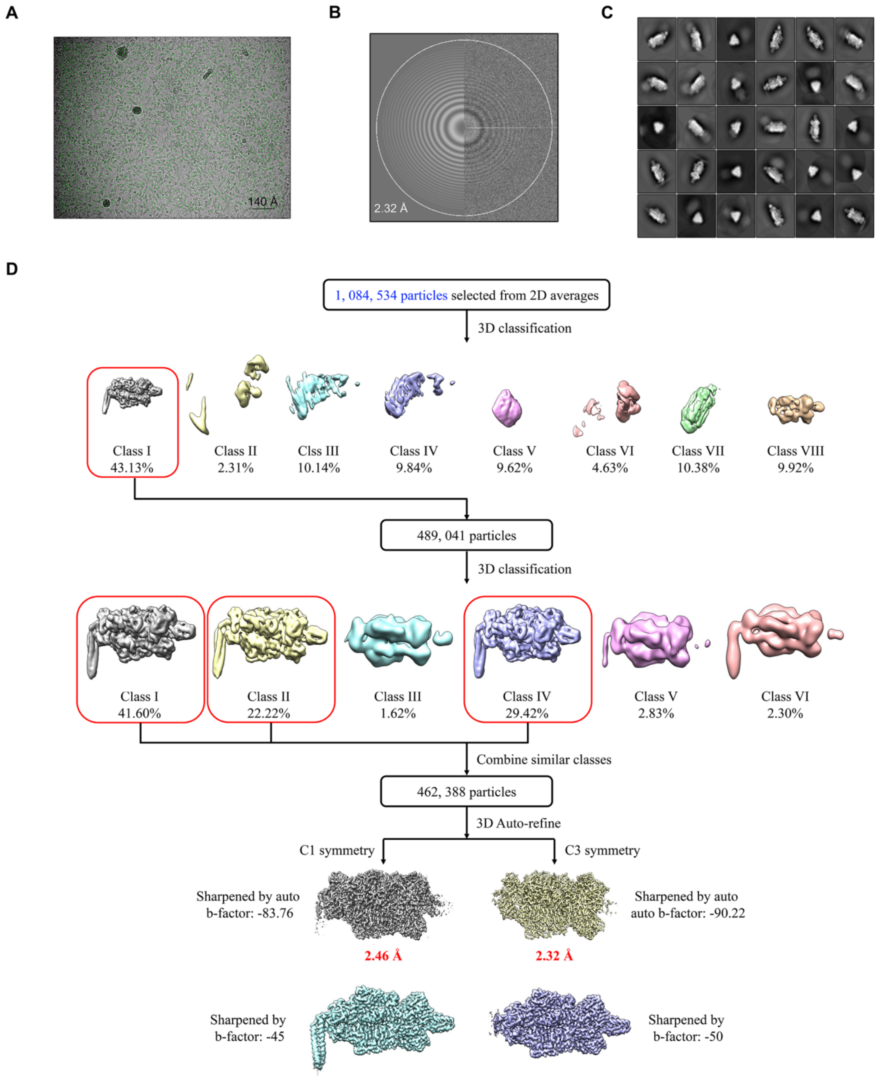

FIG S3 Cryo-EM data processing and 3D reconstruction. (A) Representative cryo-EM micrograph of Depo32 collected on a 300 kV Titan Krios equipped with a K3 direct detection detector. The Depo32 particles are indicated as green dots. (B) The rings are visible well beyond a 2.3 $Å^{-1}$ resolution. (C) Representative two-dimensional (2D) class averages for the cryo-EM particles of Depo32. The 2D class averages showed the distribution of molecules at different projection angles. (D) Flow charts of cryo-EM structure determination for Depo32. The particles from near-identical classes (red box) were combined and refined to a 2.46 Å resolution without imposing any symmetry

operation and were then further improved to a 2.32 Å resolution by applying C3 symmetry.

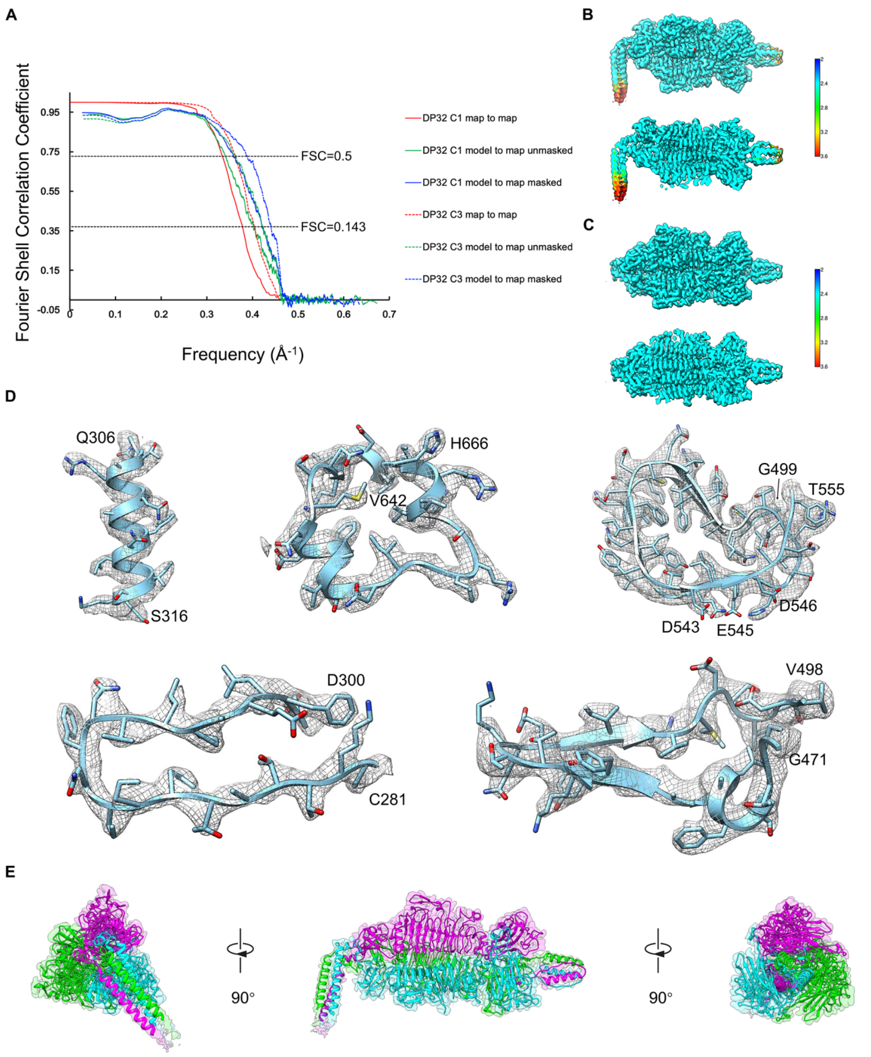

FIG S4 Refinement of the Depo32 trimer. (A) The Fourler shell correlation (FSC) curves of nonsymmetrized and C3-symmetrized maps of Depo32. Half map/half map FSC curves with labelled resolutions at FSC = 0.143, model/map FSC curves with labelled resolutions at FSC = 0.5. The local resolution of (B) nonsymmetrized and (C) C3-symmetrized maps was estimated using ResMap by the two half maps. All reported resolutions were in accordance with the gold-standard refinement procedures and the Fourier shell correlation (FSC) = 0.143 criterion. (D) Representative regions of the 2.32 Å high-resolution C3-symmetrized map superimposed with the final models. (E)

Superposition of the nonsymmetrized cryo-EM map and the atomic model of Depo32 that is built into the 2.46 Å-resolution map. From left to right, top, side, and bottom views.

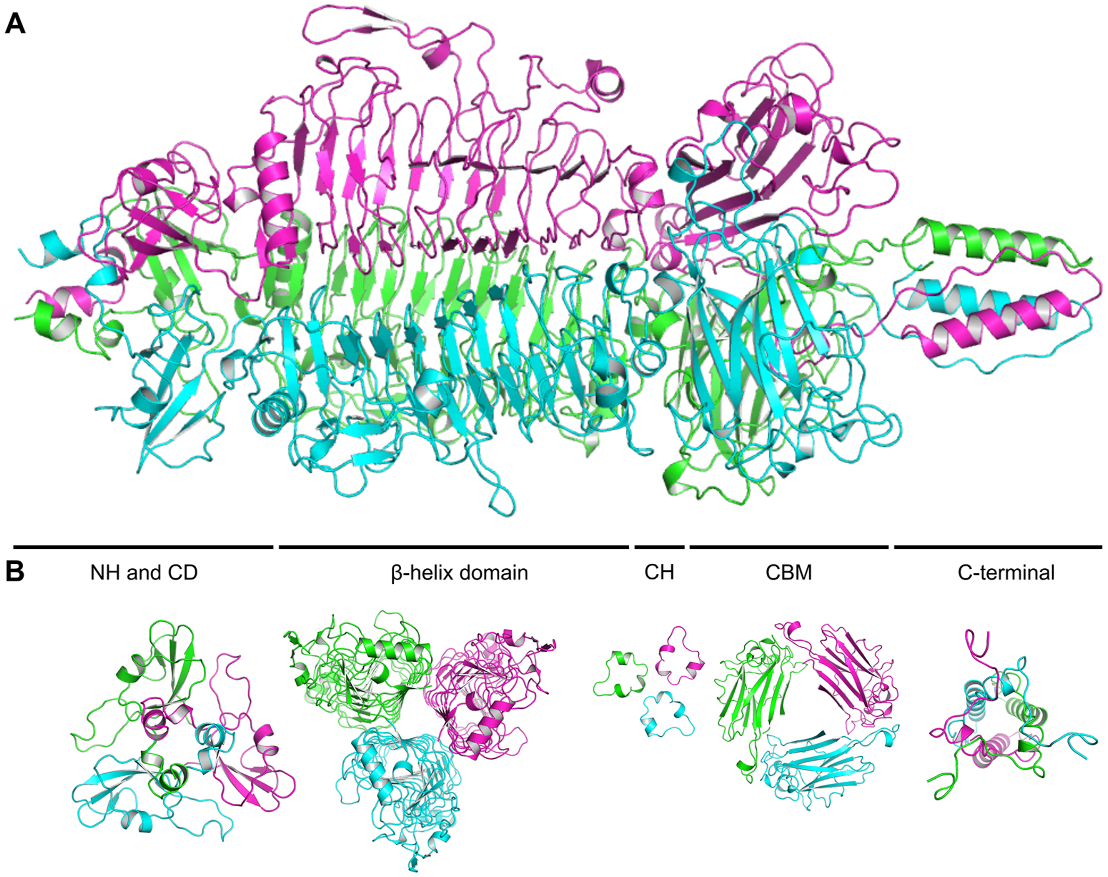

FIG S5 Structure of the Depo32 trimer and domains. (A) Atomic model of the Depo32 trimer built based on the 2.32 Å C3-symmetrized map. (B) Top view of the cartoon representation of the isolated domain trimer in (A), including the neck helix (NH) and the connection domain (CD), the β-helix domain, the connection helix domain (CH), the carbohydrate-binding module (CBM), and the C-terminal domain.

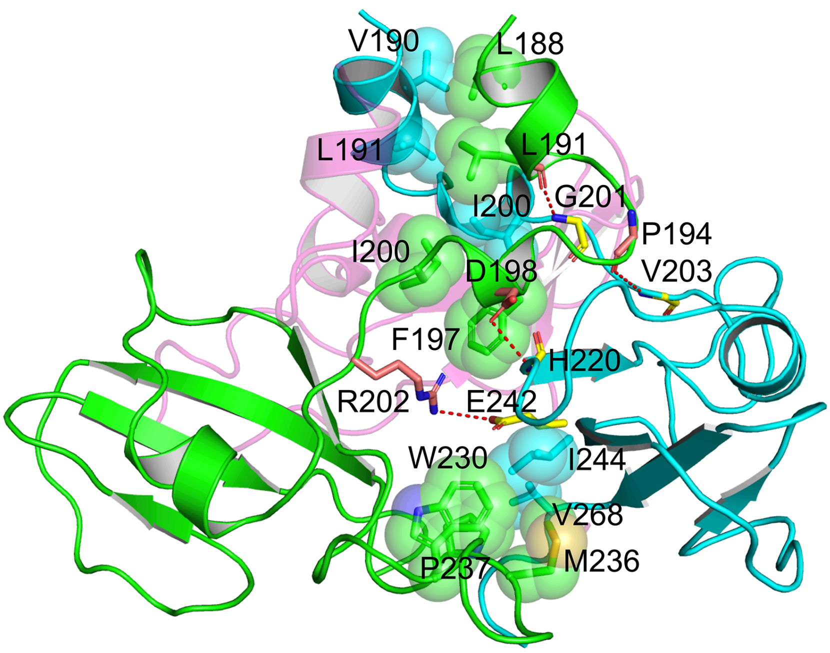

FIG S6 Interaction between two subunits on the neck helix (NH) and the connection domain (CD) of Depo32. Cartoon representation of the detailed interactions between one subunit (green) and another (cyan) on the neck helix and the subsequent connection domain of Depo32, the residues that mediate polar interactions are shown by sticks (salmon and yellow) and the red dashed lines indicate the potential hydrogen bonds formed between atoms. The residues that mediate hydrophobic interactions are shown by sticks and semitransparent spheres (green and cyan). Since the Depo32 molecule is a homotrimer, only the interactions between chain A and chain B are displayed here and the interaction between chain A and chain C as well as chain B and chain C is identical to chain A and chain B.

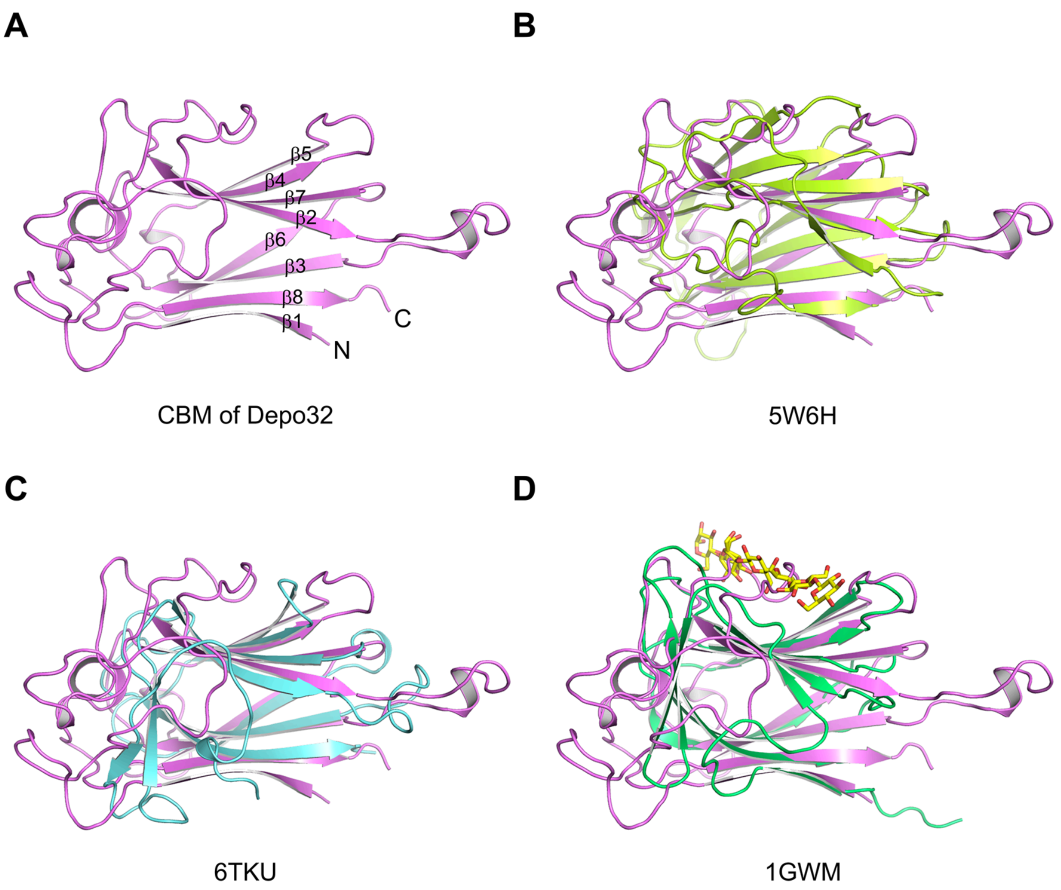

FIG S7 The structure of the carbon hydrate binding module (CBM). (A) The structure of the CBM monomer is shown by a ribbon. The CBM of Depo32 consists of a "jelly roll" folded β sandwich domain with eight antiparallel β-strands that are arranged in two layers of four-stranded β-sheets. Superposition of CBM of Depo32 with (B) TSP4 (PDB ID: 5W6H) from bacteriophage CBA120, (C) TSP (PDB ID: 6TKU) from bacteriophage KP32p38, and (D) CBM-29 (PDB ID: 1GWM) of NPC1 from plant *Piromyces equi*.

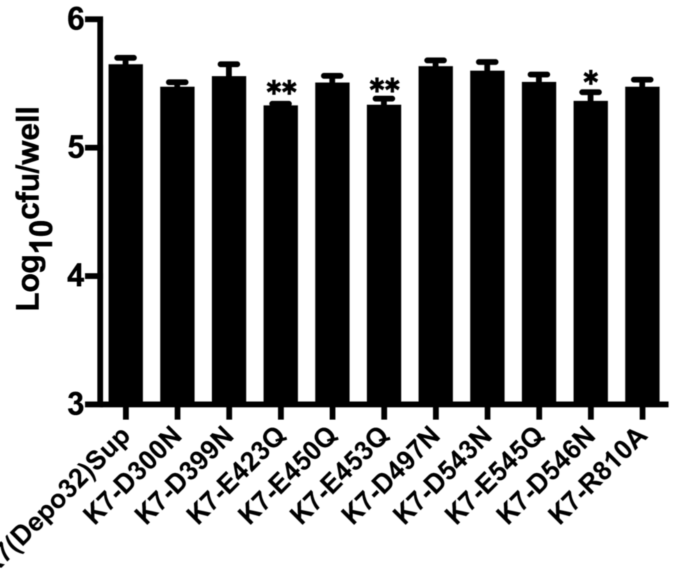

FIG S8 Endocytosis effects of RAW264.7 cells after incubation with *K. pneumoniae* K7 in the presence of Depo32-mutant proteins. Cells (5 × 10^5 cells per well) were incubated with K7 in the presence of Depo32-mutant proteins (Asp300Asn, Asp399Asn, Glu423Gln, Glu450Gln, Glu453Gln, Asp497Asn, Asp543Asn, Glu545Gln, Asp546Asn and Arg810Ala) in the supernatant at 37 °C for 1 h. After gentamicin treatment for 1 h and cell lysis, the bacterial loads in each cell sample were determined by plating. ∗ and ∗∗, significant differences at $P < 0.05$ and $P < 0.01$, respectively. Data represent the mean ± SEM of triplicate experiments.

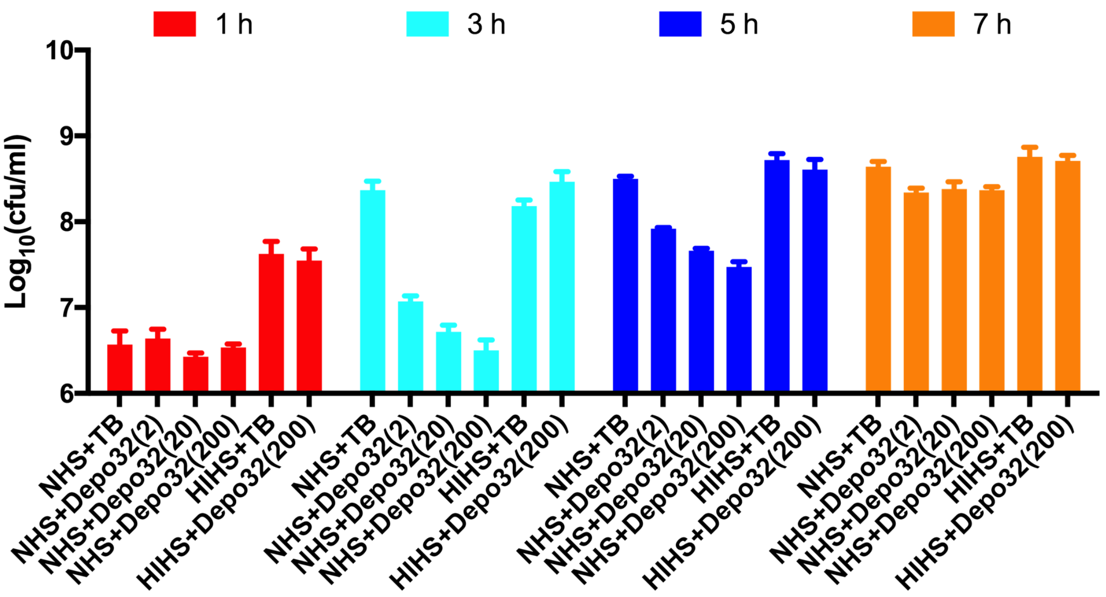

FIG S9 Bacterial loads of Depo32-treated *K. pneumoniae* with serum killing. *K. pneumoniae* K7 was mixed with different concentrations of Depo32. Depo32(2), Depo32(20) and Depo32(200) refer to 2 μg/ml, 20 μg/ml and 200 μg/ml Depo32, respectively, and Tris buffer (TB) served as a control. Then, the mixtures were further mixed with NHS or HIHS at a ratio of 1:1 (*v/v*) and incubated at 37 °C. Colonies were counted by plating at 1 h, 3 h, 5 h and 7 h.

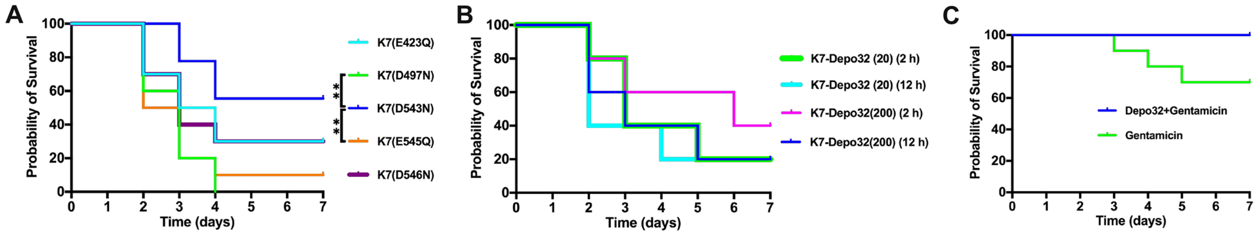

FIG S10 Survival rate. (A) *K. pneumoniae* K7 was grown to exponential phase (OD_{600} ≈ 0.6-0.8) and then treated with Depo32-mutant proteins (Glu423Gln, Asp497Asn, Asp543Asn, Glu545Gln and Asp546Asn) at 37 °C for 3 h at a final concentration of 10 μg/ml. Mice were then challenged intranasally with 1.0×10^7 CFU/mouse of K7(E423Q), K7(D497N), K7(D543N), K7(E545Q) and K7(D546N). The survival rates of these mice were determined (n = 10). (B) At 2 h or 12 h after K7 challenge, mice were treated with 200 μg (K7-Depo32 (200) group) or 20 μg (K7-Depo32 (20) group) of Depo32 at a single dose. The survival rates of mice in the K7-Depo32 (200) and K7-Depo32 (20) groups were determined (n = 5). (C) After K7 challenge, one group of mice was treated with gentamicin (1.5 mg/kg) at 1 h of infection, another group of mice was treated with Depo32 (20 μg) and gentamicin (1.5 mg/kg) at 1 h and 2 h of infection, respectively. The survival rates of these mice were determined (n = 10). Kaplan-Meier method ($P < 0.0001$, log-rank (Mantel-Cox) test) was used for statistical analysis.

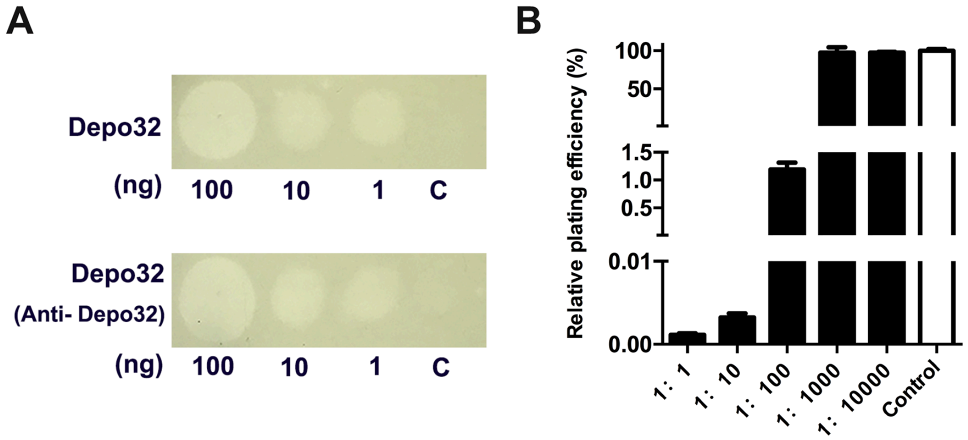

FIG S11 The effect of neutralizing antibody on Depo32. (A) Depo32 (1 mg/mL) was mixed with neutralizing antibody (0.3 mg/mL) or Tris buffer (control) in equal volumes and was incubated at 37 °C for 1 h. Then, Depo32 solutions were serially diluted and spotted on the lawns of *K. pneumoniae* K7 (1-100 ng). (B) Neutralization effect of Depo32-neutralizing antibody on the infection efficiency of K7 by phage GH-K3. The dilution rates of the antibody are indicated on the x-axis. A phage sample treated that was with Tris buffer was used as a control. Data represent the mean ± SEM of triplicate experiments.

TABLE S1 Cryo-EM data collection, processing, refinement and validation statistics

Data collection and processing		
**Microscope**	Titan Krios	
**Number of Micrographs**	773	
**Magnification**	81,000	
**Voltage (kV)**	300	
**Pixel size (Å)**	1.087	
**Defocus range (mm)**	-2.0 ~ -1.5	
**Electron exposure (e^-/Å^2)**	50	
**Dose per frame (e^-/Å^2/frame)**	1.56	
**Dose rate (e^-/pixel/s)**	23	
**Exposure time (s)**	2.56	
**Initial particle images (No.)**	1,767,920	
**Final particle images (No.)**	462,388	
Map reconstruction		
**Symmetry imposed**	C1	C3
**EMD ID**	32219	32215
**Map resolution (Å)**	2.46	2.32
**Map-sharpening B factor (Å^2)**	-83.76	-90.22
**FSC threshold**	0.143	
**Map resolution range (Å)**	8.82-2.37	9.38-2.25
Refinement		
**PDB ID**	7VZ3	7VYV
**Model resolution (Å)**	2.46	2.32
**FSC threshold**	0.5	
**Model resolution range (Å)**	196.77-2.46 (5.93-2.46)	278.27-2.32 (5.59-2.32)
Model composition		
**Nonhydrogen atoms**	17,415	16,698
**Protein residues**	2,256	2,160

**Number of Reflections**	3,030,756	3,613,319
**B factors (Protein, Å^2)**	164.01	134.29
R. M. S. deviations		
**Bond lengths (Å)**	0.010	0.009
**Bond angles (°)**	1.121	1.100
Validation		
**MolProbity score**	1.58	1.63
**Clashscore**	4.02	4.89
**Poor rotamers (%)**	0	0
**Cb outliers (%)**	0	0
Ramachandran plot		
**Favored (%)**	94.22	94.52
**Allowed (%)**	5.73	5.48
**Disallowed (%)**	0.04	0.00

TABLE S2 Primers for the construction of site-directed mutant plasmids

	Forward primer (5'-3')	Reverse primer (5'-3')
D300N	CCTGCTGAACTTTTTAACAACACAGCAAGGATG	CATCCTTGCTGTGTTGTTAAAAAGTTCAGCAGG
S331A	GAACATACTATTTTGAAAGTgCAGATACATTAAGAATAACA	TGTTATTCTTAATGTATCTGcACTTTCAAAATAGTATGTTC
E329Q	GCGCAGGAACATACTATTTTCAGAGTTCAGATACATTAAGAAT	ATTCTTAATGTATCTGAACTCTGAAAATAGTATGTTCCTGCGC
D394N	CATATTTAGGGAAGGGGATAAACTTTAACAGAAGCGTAGGAGACAATAG<br>GAAATTAG	CTAATTTCCTATTGTCTCCTACGCTTCTGTTAAAGTTTATCCCCTT<br>CCCTAAATATG
D399N	CTTTGATAGAAGCGTAGGAAACAATAGGAAATTAGTATTGG	CCAATACTAATTTCCTATTGTTTCCTACGCTTCTATCAAAG
D411N	GGAGCACGTCTATGTAAACACGTTCAGATGGGGTTTC	GAAACCCCATCTGAACGTGTTTACATAGACGTGCTCC
E421Q	GGTTTCTATGTTGGCCAACCTGAATGTATCAATC	GATTGATACATTCAGGTTGGCCAACATAGAAACC
E423Q	GTTTCTATGTTGGCGAACCTCAATGTATCAATCAGATTG	CAATCTGATTGATACATTGAGGTTCGCCAACATAGAAAC
E446Q	CAAGGTATATTCATTCAGTCTTTTAAAGAAGGGCAG	CTGCCCTTCTTTAAAAGACTGAATGAATATACCTTG
E450Q	CATTGAGTCTTTTAAACAAGGGCAGGAATATGGAC	GTCCATATTCCTGCCCTTGTTTAAAAGACTCAATG
E453Q	GAGTCTTTTAAAGAAGGGCAGCAGTATGGACATAGCGCACCAGTA	TACTGGTGCGCTATGTCCATACTGCTGCCCTTCTTTAAAAGACTC
D493N	GAATATATCAAAGTAATGAACAGTGTTAATGATGTTGGT	ACCAACATCATTAACACTGTTCATTACTTTGATATATTC
D497N	GGCTAGTGTTAATAATGTTGGTTGCCAGGCTTATTTC	GAAATAAGCCTGGCAACCAACATTATTAACACTAGCC
D543N	CATTCATAATTTACGGCACTAACCTGGAGGACATTAATGGCTT	AAGCCATTAATGTCCTCCAGGTTAGTGCCGTAAATTATGAATG
E545Q	GGCACTGATCTGCAGGACATTAATGGCTTCACAAC	GTTGTGAAGCCATTAATGTCCTGCAGATCAGTGCC
D546N	GCACTGCTCTGGAGAACATTAATGGCTTCACAAC	GTTGTGAAGCCATTAATGTTCTCCAGAGCAGTGC
D553N	GGCTTCACAACTAACGGAACAGCAATAAC	GTTATTGCTGTTCCGTTAGTTGTGAAGCC

	CAACTGACGGAACAGCAATAACGGCTAACAATATTGATACTATTGAGTC	GTAATTTGACTCAATAGTATCAATATTGTTAGCCGTTATTGCTGT
D560N	AAATTAC	TCCGTCAGTTG
D563N	GCAATAACGGCTGATAATATTAACACTATTGAGTCAAATTACTTA	TAAGTAATTTGACTCAATAGTGTTAATATTATCAGCCGTTATTGC
E566Q	CTGATAATATTGATACTATTCAGTCAAATTACTTAAAGGATAT	ATATCCTTTAAGTAATTTGACTGAATAGTATCAATATTATCAG
D572N	TGAGTCAAATTACTTAAAGAATATTTCTGGCGCTGCAATAG	CTATTGCAGCGCCAGAAATATTCTTTAAGTAATTTGACTCA
D619N	GATTGGTGGGTTCACTCCAAACGAGGCATTAAAATATAAC	GTTATATTTTAATGCCTCGTTTGGAGTGAACCCACCAATC
E620Q	GTGGGTTCACTCCAGCACAGGCATTAAAATATAACG	CGTTATATTTTAATGCCTGTGCTGGAGTGAACCCAC
H671L	TTGATGTTTCATCCCTTATTCTTAATGAAACATCACAAATTAT	ATAATTTGTGATGTTTCATTAAGAATAAGGGATGAAACATCAA
N672A	ATGTTTCATCCCTTATTCACGCAGAAACATCACAAATTATAGG	CCTATAATTTGTGATGTTTCTGCGTGAATAAGGGATGAAACAT
N687A	TAACACCAAGCACTGGTTCAGCAGTCCCTCACACAAGAATAAT	ATTATTCTTGTGTGAGGGACTGCTGAACCAGTGCTTGGTGTTA
H690L	GCACTGGTTCAAATGTCCCTCTTACAAGAATAATGTGGAGCAA	TTGCTCCACATTATTCTTGTAAGAGGGACATTTGAACCAGTGC
R692E	GTTCAAATGTCCCTCACACAGAGATAATGTGGAGCAATGGAGC	GCTCCATTGCTCCACATTATCTCTGTGTGAGGGACATTTGAAC
N697A	ACACAAGAATAATGTGGAGCGCAGGAGCAATGTATAGTTCAAC	GTTGAACTATACATTGCTCCTGCGCTCCACATTATTCTTGTGT
N707A	TGTATAGTTCAACTGACTTGGCAAACGGTTTCAGGCTTAATTA	TAATTAAGCCTGAAACCGTTTGCCAAGTCAGTTGAACTATACA
R711E	CTGACTTGAACAACGGTTTCGAGCTTAATTATCTAAGCAACCA	TGGTTGCTTAGATAATTAAGCTCGAAACCGTTGTTCAAGTCAG
Y714A	CGGTTTCAGGCTTAATGCTCTAAGCAACCATAACG	CGTTATGGTTGCTTAGAGCATTAAGCCTGAAACCG
N719A	TTAATTATCTAAGCAACCATgcaGAACCGCTTACACCTATGCA	TGCATAGGTGTAAGCGGTTCtgcATGGTTGCTTAGATAATTAA
P724A	ACCATAACGAACCGCTTACAgcaATGCATCTATACAATGAGTT	AACTCATTGTATAGATGCATtgcTGTAAGCGGTTCGTTATGGT
Y728A	GCTTACACCTATGCATCTAGCTAATGAGTTTTCTGTTTC	GAAACAGAAAACTCATTAGCTAGATGCATAGGTGTAAGC
E735N	ACAATGAGTTTTCTGTTTCTAATTTTGGAGGATCAGTAACAGA	TCTGTTACTGATCCTCCAAAATTAGAAACAGAAAACTCATTGT
E748N	CAGAATCAAACGCCTTGGATAATATTAAATACATATTCATTCA	TGAATGAATATGTATTTAATATTATCCAAGGCGTTTGATTCTG
K750E	CAAACGCCTTGGATGAAATTGAGTACATATTCATTCAAACGAC	GTCGTTTGAATGAATATGTACTCAATTTCATCCAAGGCGTTTG

Q755A	AAATTAAATACATATTCATTGCAACGACTTATGCAAACTCAGG	CCTGAGTTTGCATAAGTCGTTGCAATGAATATGTATTTAATTT
R765A	CAAACTCAGGTGATGGGGCGTTTATAATTCAGGCGCTTG	CAAGCGCCTGAATTATAAACGCCCCATCACCTGAGTTTG
Q769A	GATGGGAGGTTTATAATTGCTGCGCTTGATGCCAGTG	CACTGGCATCAAGCGCAGCAATTATAAACCTCCCATC
R799A	TCCCGATAAGTGGATTCGTTGAGTTTGATGTTCCGACAGGTGC	GCACCTGTCGGAACATCAAACTCAACGAATCCACTTATCGGGA
K808E	ATGTTCCGACAGGTGCGAAGGAGATAAGATATGGATTTGTTAA	TTAACAAATCCATATCTTATCTCCTTCGCACCTGTCGGAACAT
R810A	CGACAGGTGCGAAGAAAATAGCATATGGATTTGTTAACAGTGC	GCACTGTTAACAAATCCATATGCTATTTTCTTCGCACCTGTCG
R824E	CCAATTACACTGGCTCACTTGAGTCGCACTTCATGTCTGGTTT	AAACCAGACATGAAGTGCGACTCAAGTGAGCCAGTGTAATTGG
N834A	TCATGTCTGGTTTTGCATATGCAAAAAGGTTCTTCCTTAAAAT	ATTTTAAGGAAGAACCTTTTTGCATATGCAAAACCAGACATGA
K835E	TGTCTGGTTTTGCATATAATGAGAGGTTCTTCCTTAAAATATA	TATATTTTAAGGAAGAACCTCTCATTATATGCAAAACCAGACA
R836E	CTGGTTTTGCATATAATAAAGAGTTCTTCCTTAAAATATATGC	GCATATATTTTAAGGAAGAACTCTTTATTATATGCAAAACCAG
K840E	ATAATAAAAGGTTCTTCCTTGAGATATATGCTGTATACAATGA	TCATTGTATACAGCATATATCTCAAGGAAGAACCTTTTATTAT
Y842F	AAAGGTTCTTCCTTAAAATATTTGCTGTATACAATGACTTAGG	CCTAAGTCATTGTATACAGCAAATATTTTAAGGAAGAACCTTT

122

TABLE S3 Grouping and treatment of mice in this study

Survival rate

Groups	Doses [1]	Times [2]	N [3]
K7			5
K7(Depo32)			5
K7-Depo32 (200)	200 µg	1 h	5
K7-Depo32 (200)	200 µg	2 h	5
K7-Depo32 (200)	200 µg	12 h	5
K7-Depo32 (20)	20 µg	1 h	5
K7-Depo32 (20)	20 µg	2 h	5
K7-Depo32 (20)	20 µg	12 h	5
K7-Depo32 (20S)	20×3 µg	1 h, 25 h, 49 h (3 days)	5
Gentamicin	1.5 mg/kg	1 h	10
Depo32 + Gentamicin	20 µg + 1.5 mg/kg	1 h, 2 h	10
K7(E423Q)			10
K7(D497N)			10
K7(D543N)			10
K7(E545Q)			10
K7(D546N)			10

Bacterial loads, histopathological damage, and cytokine expression

Groups	Doses [1]	Times [2]	N [3]
K7			15
K7(Depo32)			25
K7-Depo32 (200)	200 µg	1 h	25
K7-Depo32 (20)	20 µg	1 h	25
K7-Depo32 (20S)	20×3 µg	1 h, 25 h, 49 h (3 days)	25

(*Continued*)

TABLE S3 Continued

Neutralizing antibody on the therapeutic efficacy			
**Groups**	**Doses [1]**	**Times [2]**	**N [3]**
K7 (Survival rate)			10
K7+Depo32 (200) (Survival rate)	200 μg		10
K7			15
K7+Depo32 (200)	200 μg	1 h	25

[1] Doses: the doses of Depo32 or gentamicin for intranasal treatment.

[2] Times: the time after *K. pneumoniae* infection.

[3] N: the number of mice in each group.

MATERIALS AND METHODS

Capsule staining

Bacterial solutions (100 μl) of K7 and K7(Depo32) were thoroughly mixed with an equal volume of 1% aqueous Congo red solution (Sigma–Aldrich, St. Louis, MO, United States) for 1 min, and 5 μl of the mixture was uniformly coated on a glass slide and air-dried to form a film. The film areas were then gently coated with 10 μl of Maneval's solution (3.33% phenol, 4.44% glacial acetic acid, 2.67% ferric chloride, 0.02% acid fuchsin; Sigma–Aldrich, St. Louis, MO, United States) and were air-dried. Visualization and imaging were performed using an optical microscope imaging system (100×, oil; Olympus CX-41; Olympus America, Center Valley, PA 18034-0610, United States).

Scanning electron microscopy

After three washes with sterile PBS, K7 and K7(Depo32) were immobilized with 4% glutaraldehyde overnight. Bacterial cells were dehydrated with different concentrations (20%, 50%, 70%, 90%, and 100%) of ethanol and were subsequently freeze-dried on cover glasses and observed through scanning electron microscopy (SEM) (Hitachi S-3400N, Hitachi High-Technologies Europe GmbH, Krefeld, Germany).

CPS analysis

As previously described (1), a mucoviscosity assay was used to detect the relative content of *K. pneumoniae* capsules. Briefly, *K. pneumoniae* strains with or without Depo32 treatment (100 μg/ml) were centrifuged at 10,000 ×*g* for 5 min and were

washed twice with PBS. The concentrations of all strains were set at OD_{600}~1.0. After centrifugation at 1,000 × g for 5 min, the optical density of the supernatant at 600 nm (OD_{600}) was measured using a microplate reader (Bio–Rad, CA, United States).

Extraction and purification of CPS from the *K. pneumoniae* strains with or without Depo32 treatment (100 μg/ml) were performed as previously described (2). The purified CPSs were precipitated by adding cetylpyridinium chloride (CPC) to a final concentration of 5 mg/mL, and then the OD_{600} was measured after 10 min of incubation at 25 °C. In addition, CPS phenotypes were analysed by alcian blue staining (3).

Serum sensitivity assay

Normal human serum (NHS) was obtained from volunteers at the First Hospital of Jilin University. According to a previously described method (4), *K. pneumoniae* K7 (exponential phase, final concentration 1×10^6 CFU/ml) was mixed with Depo32 (final concentration 2 μg/ml, 20 μg/ml or 200 μg/ml), mixed with NHS or heat-inactivated human serum (HIHS) at a ratio of 1:1 (*v/v*) and incubated at 37 °C. Colonies were counted on LB plates at 1 h, 3 h, 5 h and 7 h.

Effect of neutralizing antibody on Depo32

A neutralizing antibody against Depo32 (concentration: 0.3 mg/ml, titer: 1:128,000) was made by PolyExpress™ at GenScript Biological Technology Co., Ltd. (antibody titer: 1:128,000; Nanjing, China). Phage plating efficiencies were determined to evaluate the ability of the Depo32-neutralizing antibody to inhibit the infection of phage GH-K3 on the host strain. In brief, the neutralizing antibody (0.3 mg/ml) was serially diluted by 10-fold, and 100 μl of phage GH-K3 (1×10^{10} PFU) samples were

mixed with the same volume of diluted antibodies and were incubated at 37 °C for 1 h. The number of activated phages that remained in the mixtures was determined by a plaque assay in triplicate.

Depo32 (1 mg/ml) was mixed with neutralizing antibody (0.3 mg/mL) in equal volumes and was incubated in a 37 °C water bath for 1 h. The enzyme activity was detected by spot assays after serial dilution as described above.

References

1. Bachman MA, Breen P, Deornellas V, Mu Q, Zhao L, Wu W, Cavalcoli JD, Mobley HL. 2015. Genome-wide identification of *Klebsiella pneumoniae* fitness genes during lung infection. mBio 6:e00775. http://doi.org/10.1128/mBio.00775-15
2. Feldman MF, Mayer Bridwell AE, Scott NE, Vinogradov E, McKee SR, Chavez SM, Twentyman J, Stallings CL, Rosen DA, Harding CM. 2019. A promising bioconjugate vaccine against hypervirulent *Klebsiella pneumoniae*. Proc Natl Acad Sci U S A 116:18655-18663. http://doi.org/10.1073/pnas.1907833116
3. Møller HJ, Heinegård D, Poulsen JH. 1993. Combined alcian blue and silver staining of subnanogram quantities of proteoglycans and glycosaminoglycans in sodium dodecyl sulfate-polyacrylamide gels. Anal Biochem 209:169-175. http://doi.org/10.1006/abio.1993.1098
4. Majkowska-Skrobek G, Latka A, Berisio R, Squeglia F, Maciejewska B, Briers Y, Drulis-Kawa Z. 2018. Phage-borne depolymerases decrease *Klebsiella pneumoniae* resistance to innate defense mechanisms. Front Microbiol 9:2517. http://doi.org/10.3389/fmicb.2018.02517
